# Exposure to bacterial PAMPs before RSV infection exacerbates innate inflammation and disease via IL-1α and TNF-α

**DOI:** 10.1016/j.mucimm.2024.08.002

**Published:** 2024-12

**Authors:** Amber R. Owen, Ana Farias, Anne-Marie Levins, Ziyin Wang, Sophie L. Higham, Matthias Mack, John S. Tregoning, Cecilia Johansson

**Affiliations:** a Respiratory Infections, National Heart and Lung Institute, Imperial College London, United Kingdom; b Department of Infectious Disease, Imperial College London, United Kingdom; c Department of Nephrology, University Hospital Regensburg, Regensburg, Germany

**Keywords:** Respiratory infections, Virus infections, Pro-inflammatory cytokines, Innate immunity, Bacteria

## Abstract

Respiratory syncytial virus (RSV) can cause severe lower respiratory tract infections. Understanding why some individuals get more serious disease may help with diagnosis and treatment. One possible risk factor underlying severe disease is bacterial exposure before RSV infection. Bacterial exposure has been associated with increased respiratory viral-induced disease severity but the mechanism remains unknown. Respiratory bacterial infections or exposure to their pathogen associated molecular patterns (PAMPs) trigger innate immune inflammation, characterised by neutrophil and inflammatory monocyte recruitment and the production of inflammatory cytokines. We hypothesise that these changes to the lung environment alter the immune response and disease severity during subsequent RSV infection. To test this, we intranasally exposed mice to LPS, LTA or *Acinetobacter baumannii* (an airway bacterial pathogen) before RSV infection and observed an early induction of disease, measured by weight loss, at days 1–3 after infection. Neutrophils or inflammatory monocytes were not responsible for driving this exacerbated weight loss. Instead, exacerbated disease was associated with increased IL-1α and TNF-α, which orchestrated the recruitment of innate immune cells into the lung. This study shows that exposure to bacterial PAMPs prior to RSV infection increases the expression of IL-1α and TNF-α, which dysregulate the immune response resulting in exacerbated disease.

## Graphical abstract

(1) Intranasal exposure to bacterial PAMPs increases the expression of cytokine and chemokines, in particular (2) IL-1α and TNF-α. (3) The expression of cytokines and chemokines drives the recruitment of innate immune cells including neutrophils and inflammatory monocytes into the lung. (4) When mice are infected 12h later with RSV this further (5) promotes the expression of IL-1α and TNF-α in the lung. (6) resulting in exacerbated disease. Illustration made in Biorender.

**Figure:**
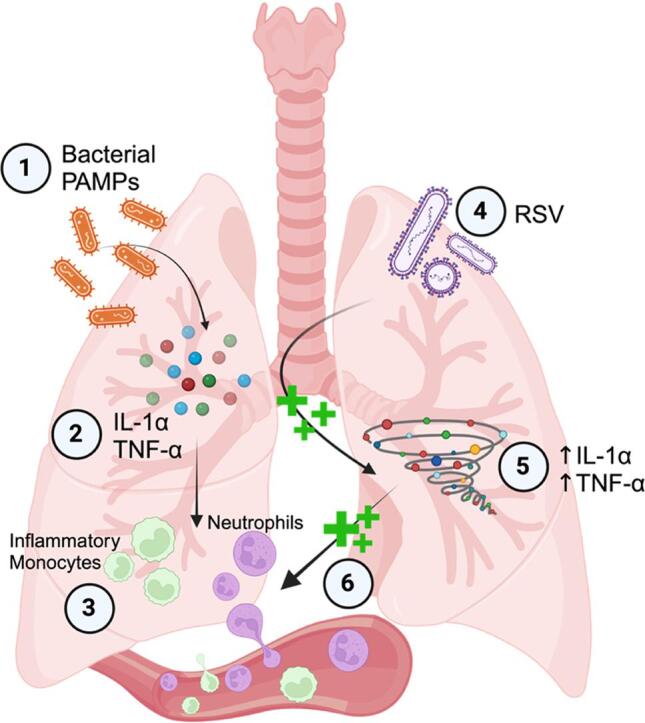

## Introduction

Respiratory viral infections are a prevalent cause of morbidity and mortality worldwide.^1^ Infection with Respiratory Syncytial Virus (RSV) is common and reoccurs throughout life.^2^ In healthy adults, infection with RSV will typically result in mild upper airway symptoms.^3^ However, RSV has the propensity to cause severe lower respiratory tract infections in some people^3^; the most vulnerable populations are infants and the elderly.^4^ By the age of 2 years, over 90 % of infants have been infected with RSV, with 1–3 % of cases resulting in hospitalisation, making RSV one of the leading causes of global infant mortality by communicable disease.^1^, ^4^ Likewise, RSV is an important cause of disease and death in the elderly.^5^ Before the emergence of SARS-CoV-2 and the COVID-19 pandemic, RSV accounted for approximately 6 % of all respiratory disease associated deaths in adults over the age of 65, resulting in over 8000 deaths in the UK per annum.^5^ Despite the enormous global burden of RSV, vaccines have only recently become available for use in elderly patients and pregnant women.^6^, ^7^, ^8^ Phase III clinical trials have demonstrated good efficacy in both elderly recipients and the newborn infants of pregnant recipients.^7^, ^8^ These novel vaccines are now licensed and are likely to be widely used going forward. However, data on the long-term efficacy of these vaccines are not yet available and not everyone will get vaccinated. Therefore, understanding the immune determinants that predispose individuals to more severe disease is useful in further developing diagnostics, directing treatments and preventing hospitalisations.

Much of the disease following RSV infection is driven by an inappropriate or excessive immune response.^9^ In mouse models, disease severity can be measured by monitoring weight loss.^10^, ^11^, ^12^ Following RSV infection, it is typical for mice to lose significant weight beginning on day 5 post infection followed by recovery by day 8 post infection.^10^, ^11^, ^12^ This weight loss is caused by appetite loss, a common symptom in children with severe RSV infection, associated with lower body weight at hospital admission.^13^, ^14^, ^15^ Previous studies have focused on the role of the adaptive immune response in driving this weight loss.^10^, ^11^, ^12^ However, the innate immune response can also play a role in increasing symptom severity both directly and indirectly by shaping the resulting adaptive immune response. For example, neutrophils and monocytes are detected in the lungs early during RSV infection in both humans and mice.^11^, ^16^, ^17^, ^18^, ^19^, ^20^, ^21^ They are frontline innate immune cells that migrate in large numbers to infected tissues via the gradient of chemokines.^16^, ^22^, ^23^, ^24^ Once at the site of infection, neutrophils perform a wide range of antimicrobial mechanisms, including the release of degradative enzymes or antimicrobial peptides stored in their granules, phagocytosis, production of reactive oxygen species and release of neutrophil extracellular traps.^19^ Whilst, these mechanisms are effective against pathogens they can damage host cells and contribute to pathology in a number of respiratory diseases.^19^, ^25^, ^26^ The contribution of neutrophils in RSV-induced disease appears to depend on their recruitment kinetics; they do not contribute to disease severity during RSV infection,^10^, ^27^ however their presence in the airways prior to RSV infection is associated with increased severity in both mice and humans.^27^ Inflammatory monocytes, play an important role in the early anti-viral response following RSV infection.^11^ However, they also produce a range of pro-inflammatory cytokines and death-inducing receptors such as TNF-related apoptosis-inducing ligand (TRAIL), which can drive cellular damage during influenza virus infection.^28^, ^29^

There are many environmental factors that influence the inflammatory status of the respiratory mucosa including air pollution, allergens, cigarette smoke exposure and the use of e-cigarettes.^30^, ^31^, ^32^, ^33^ However, one of the most common and well-studied factors is bacterial infection.^34^ Co-infection with bacterial respiratory pathogens is common amongst infants hospitalised with severe RSV infection. In a study of 175 children hospitalised following RSV infection, ∼21 % had confirmed bacterial co-infection and another ∼ 20 % had low grade bacterial growth, indicative of changes to the microbiota.^35^ Inflammation following bacteria exposure is initiated by pattern recognition receptor (PRR) binding to pattern associated molecular patterns (PAMPs) released by or found on the outer surface of bacteria.^36^ Arguably the most well-known PAMP is lipopolysaccharide (LPS), expressed on the surface of gram-negative bacteria.^37^ LPS is recognised by the host toll-like receptor (TLR) 4 initiating a signalling cascade via MyD88/TRIF, leading to the activation of transcription factors NF-kB and IRF3.^38^ This ultimately drives the release of cytokines and chemokines including CXCL1, CCL2, IFN-β, IL-1α, IL-1β and TNF-α.^39^, ^40^ In the majority of reported cases of bacterial co-infections with severe RSV infection, the bacterial infection was more likely acquired secondary to RSV, often following hospitalisation.^41^ However, bacterial infection, exposure to bacterial PAMPs or changes to the lung microbiota can occur before RSV infection and have the potential to exacerbate disease outcomes.^42^, ^43^ Exposure to increased levels of environmental LPS has been linked to increased cases of hospitalisation following RSV infection in children.^42^ Similarly, analysis of the microbiota in a cohort of infants, found that increased levels of gram-negative gammaproteobacteria before RSV infection was associated with increased incidence of severe disease following infection.^43^ Gammaproteobacteria are common in the environment and can cause respiratory infections. For example, *Acinetobacter baumannii* was found to be one of the most prominent organisms present in indoor dust particles, which are thought to aggravate inflammation in chronic lung disease patients.^44^ *Acinetobacter baumannii* can colonise the respiratory tract, predominantly causing disease in immunocompromised patients, but it is also enriched in the lung microbiota of asthma and COPD patients.^44^, ^45^, ^46^ Subtle changes to the microbiota or exposure to bacteria or their PAMPs before RSV infection may not be detectable as a productive infection.^47^ As a result, the mechanism of how bacterial exposure before respiratory viral infection can affect disease outcomes is largely understudied.

In this study we show that exposure to bacteria and/or their PAMPs drive inflammation of the respiratory mucosa and that it can impact RSV disease severity in mice. This increased disease severity occurs in the innate phase of the infection and is associated with increased recruitment of innate immune cells into the lungs orchestrated by IL-1α and TNF-α.

## Results

***Intranasal LPS exposure recruits neutrophils and inflammatory monocytes into the lungs and airways***

The aim of this study was to understand the role of airway pre-exposure to bacteria and bacterial PAMPs on disease severity following RSV infection. Therefore, inflammation in the lungs and airways over time following intranasal exposure to LPS was profiled. Mice received a single intranasal dose of 1 µg LPS and responses were compared to those receiving PBS. This LPS dose was selected after titration experiments demonstrating it did not induce weight loss (data not shown). After 6, 12, 18 and 24 h, mice were euthanised and the immune response was profiled in the lungs and airways (bronchoalveolar lavage; BAL) using flow cytometry (see Supplementary Fig. 1 for gating strategy and Supplementary Fig. 2a for experimental setup). Intranasal LPS drove rapid and significant recruitment of neutrophils into the lungs and airways. Total neutrophil numbers peaked at 6 h in the airways and 12 h in the lungs after LPS, after which numbers steadily declined (Supplementary Fig. 2b-c). Neutrophil recruitment followed the expression of *Cxcl1*, which peaked at 6 h after intranasal LPS exposure (Supplementary Fig. 2d). LPS also drove significant recruitment of inflammatory monocytes peaking at 12 h (Supplementary Fig. 2e), matched by expression of the monocyte chemokine *Ccl2* which peaked 6 h after intranasal LPS (Supplementary Fig. 2f). Twelve hours after intranasal LPS administration, neutrophils accounted for a large proportion (34 %) of cells in the lung and were the majority cell type (68 %) in the airways (Supplementary Fig. 2g-h).

In addition to immune cell recruitment, TLR4 activation by LPS leads to general inflammation.^40^ Therefore, we profiled how intranasal LPS altered the transcription of inflammatory cytokine genes in the lungs. LPS increased the expression of IL-1 family, pro-inflammatory cytokines *Il1a* and *Il1b* in the lungs early (6 h) after exposure (Supplementary Fig. 2i-j). Expression of *Il1b* subsequently declined across the time course, but expression of *Il1a* remained steady until 18 h after intranasal LPS, after which it then declined (Supplementary Fig. 2i-j). Intranasal LPS also led to increased expression of *Tnfa*, which peaked at 6 h then steadily declined (Supplementary Fig. 2k). Taken together, these data demonstrate the ability of LPS to initiate an inflammatory response in the lungs.

***Intranasal LPS 12 h before RSV infection induces rapid weight loss early after infection associated with increased inflammation***

**Fig. 1:**
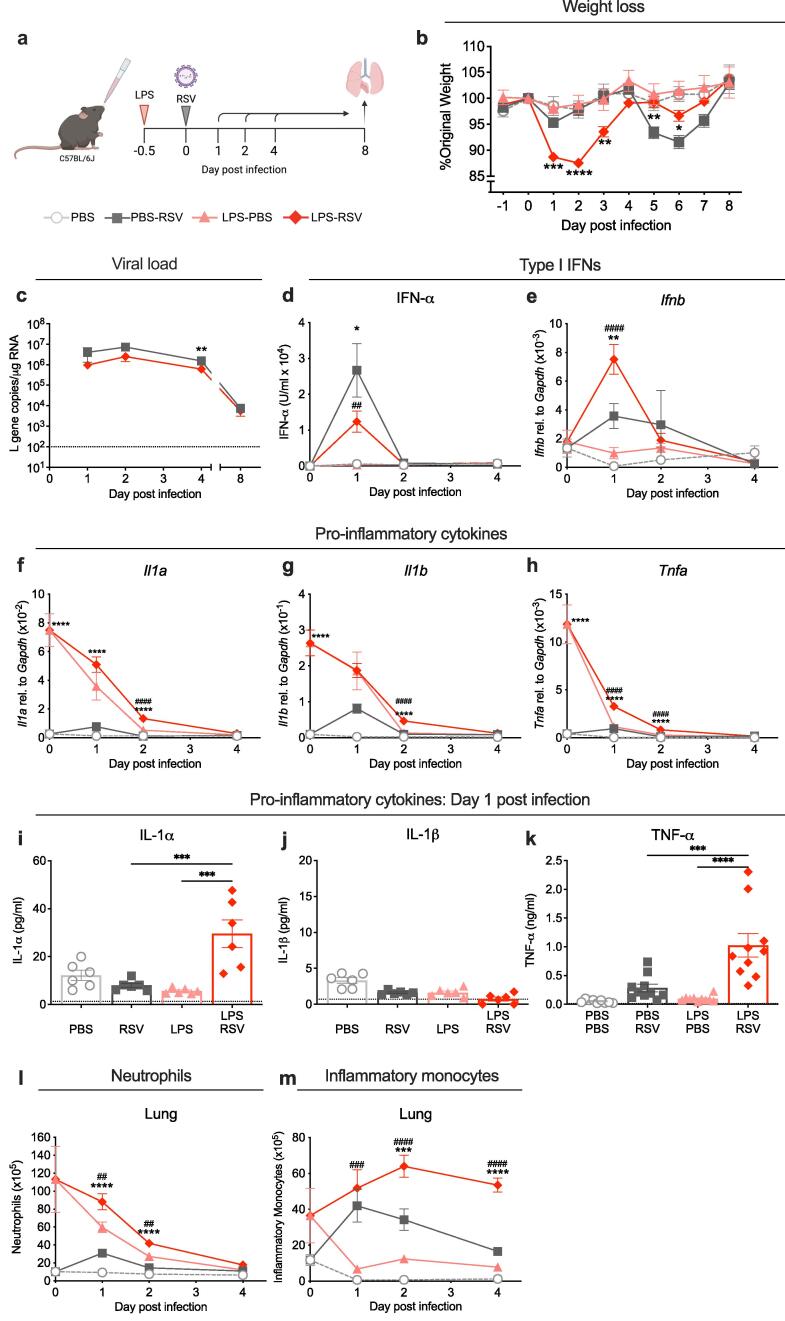
**Immune cell recruitment to the lung in mice exposed to LPS before RSV infection. a)** Graphic showing experimental design. Mice were exposed intranasally to 1 μg LPS or PBS 12 h before RSV or mock (PBS) infection (day −0.5). At days 0,1, 2, 4 or 8 after infection airway and lung cells were collected for analysis using flow cytometry, ELISA and qPCR. **b)** Daily weight of mice represented as percentage of original body mass on day 0. **c)** Viral load in the lungs represented as *L* gene copy number/μg RNA measured by qPCR and normalised to *Gapdh.***d)** Levels of IFN-α in the BAL supernatant measured by ELISA. Expression of **e)***Ifnb,***f)***Il1a*, **g)***Il1b* and **h)***Tnfa* in the lung, measured by qPCR and normalised to *Gapdh*. Levels of **i)** IL-1α and **j**) IL-1β in the BAL supernatant measured by multiplex and **k)** levels of TNF-α in the BAL supernatant measured by ELISA, on day 1 post infection. Total number of **l)** neutrophils or **m)** total number of inflammatory monocytes in the lungs over time following RSV infection quantified using flow cytometry. At days 0–4 data are pooled from 2 experiments per time point. At day 1, 2 and 4 after infection, n = 10. At day 8, PBS only and LPS only data are from 1 experiment n = 5; RSV only and LPS-RSV, data are pooled from 2 experiments n = 9. Black dotted line represents the lower limit of detection. Time courses are plotted as mean ± SEM. A one-way ANOVA with multiple comparisons test was carried out for each timepoint. Asterisks represent the *p* value for LPS-RSV data compared to RSV infected controls; *≤0.05, **≤0.01, ***≤0.001, ****≤0.0001. Hashtags represent the *p* value for LPS-RSV compared to LPS only controls; #≤0.05, ##≤0.01, ###≤0.001, ####≤0.0001.

To investigate how LPS-induced inflammation in the lungs influence RSV infection, mice were given an intranasal dose of LPS 12 h before RSV infection; this time point was chosen as the peak of innate immune cell infiltration following LPS (Supplementary Fig. 2). Mice were euthanised on days 1, 2, 4 or 8 after infection and lung and airway cells were profiled (Fig. 1a). Mice infected with RSV lost ∼ 5 % of their original body weight at day 1 post infection but quickly recovered by day 2. This weight loss was not statistically significant compared to PBS controls. At day 6 post infection, RSV infected mice experienced a second wave of more severe weight loss, peaking at ∼ 10 % loss of their original body weight, then recovering by day 8 (Fig. 1b). Mice that received LPS without subsequent RSV infection did not lose weight during the experiment (Fig. 1b). In contrast, mice that received intranasal LPS before RSV infection lost significantly more weight than RSV only mice during the first 3 days of infection. Weight loss peaked at ∼ 12 % of original body weight on day 2 post infection followed by recovery by day 4 post infection. On days 5–7, these mice lost significantly less weight than the RSV only group (Fig. 1b). In addition to weight loss, LPS before RSV infection was also associated with visible signs of illness between days 1–3 post infection including ruffled fur, hunched posture and slowed movement (data not shown).

Viral load in the lungs was quantified by measuring RSV L gene copies by qPCR. Despite the early weight loss, mice that received LPS before RSV infection had significantly lower viral load in the lungs compared to RSV only mice at day 4 post infection (Fig. 1c). This suggested that the weight loss was driven by the immune response to the virus. Therefore, cytokine expression in the lung was measured using ELISA and qPCR. IFN-α and *Ifnb* peaked at day 1 post RSV infection (Fig. 1d-e). In mice exposed to LPS before RSV infection, IFN-α in the BAL trended lower than RSV controls at day 1 post infection (Fig. 1d). However, expression of *Ifnb* was significantly increased (Fig. 1e). RSV infection also induced rapid and transient expression of *Il1a, Il1b* and *Tnfa* on day 1 post infection (Fig. 1f-h). As seen after LPS alone (Supplementary Fig. 2i-k), expression of *Il1a, Il1b* and *Tnfa* increased 12 h after LPS exposure but declined during the first 2 days of infection (Fig. 1f-h). Compared to RSV only infected mice, expression levels were higher in LPS exposed, RSV-infected mice at days 0–2 post infection (Fig. 1f-h). In mice exposed to LPS and subsequently infected with RSV, expression of *Il1a, Il1b* and *Tnfa* remained elevated for longer compared to both LPS only and RSV only mice (Fig. 1f-h). On day 2 post infection, *Il1a* and *Il1b* expression were significantly higher compared to either LPS only or RSV only mice (Fig. 1f-g). *Tnfa* expression was significantly higher at day 1 and 2 post infection compared to LPS only and RSV only mice (Fig. 1h). Corresponding with the increased gene expression of *Il1a* and *Tnfa*, IL-1α and TNF- protein in the airways was also significantly elevated on day 1 post infection in mice exposed to LPS before RSV infection compared to LPS only and RSV only mice (Fig. 1i and k). In contrast, IL-1β protein was undetectable in the airways (Fig. 1j). RSV infection has also been demonstrated to induce large production of IL-6 in the lungs,^55^ therefore we measured the effect of LPS pre-exposure on the IL-6 response to RSV infection. Whilst levels of IL-6 increased in the airways following RSV infection peaking at day 1 post infection, LPS exposure before RSV infection had no significant effect on airway IL-6 (Supplementary Fig. 3a). This suggests that LPS may have a more specific effect on the immune response towards RSV than boosting general inflammation.

The infiltration of immune cells into the lungs and airways was profiled by flow cytometry. RSV infection led to neutrophil recruitment into the lungs (Fig. 1i) and airways (Supplementary Fig. 3b), peaking on day 1 post infection. However, the total numbers of neutrophils were significantly lower than that detected following LPS only (Fig. 1l). RSV infection after LPS exposure did not further increase the number of neutrophils compared to LPS alone, but it did result in persistently higher numbers of neutrophils in the lungs at day 1 and 2 compared to RSV only or LPS only mice (Fig. 1l). In addition to neutrophils, inflammatory monocytes were recruited into the lungs (Fig. 1m) and airways (Supplementary Fig. 3c), following RSV infection peaking at day 1 and 2 post infection, respectively. LPS pre-exposure significantly increased the recruitment of inflammatory monocytes at day 2 and 4 post RSV infection (Fig. 1m). The increased recruitment of inflammatory cells in mice exposed to LPS before RSV infection was evenly distributed throughout the lung tissue on day 1 post infection determined by histology (Supplementary Fig. 3d).

T cell recruitment into the lungs was also measured on days 4 and 8 post infection. CD4^+^ T cells were increased in the lungs on day 4 post RSV infection but were not significantly altered by LPS exposure before infection (Supplementary Fig. 3e-f). By day 8 post infection, CD8^+^ T cells were increased in the lungs of RSV infected mice compared to PBS mice. Total numbers of CD4^+^ and CD8^+^ T cells trended lower in mice which received LPS 12 h before RSV infection, but this was not found to be statistically significant (Supplementary Fig. 3e-f). Overall, these data suggest that exposure to LPS before RSV infection results in sustained levels of neutrophils, monocytes and expression of pro-inflammatory cytokines.

***Intranasal exposure to Acinetobacter baumannii before RSV infection exacerbates early weight loss associated with an increased innate inflammatory response***

Next, we wanted to confirm that the LPS pre-exposure model reflects exposure/co-infection with gram-negative bacteria. Given its environmental abundance and association with chronic inflammatory lung disease, we decided to investigate how *Acinetobacter baumannii* exposure before RSV infection altered disease.^44^ Mice were intranasally infected with an antibiotic susceptible strain of *A. baumannii* (ATCC 17978) at low dose (5-8x10^5^ CFU) (Supplementary Fig. 4a). The infection dose was selected based on previous data, which demonstrated that low dose intranasal infection with ATCC 17978 resulted in minimal weight loss and was cleared rapidly from the lungs and airways.^48^ Bacterial load quickly declined following infection suggesting intranasal exposure to *A. baumannii* resulted in short-lived, non-productive infection (Supplementary Fig. 4b-c). Despite the rapid clearance, *A. baumannii* infection resulted in neutrophil and inflammatory monocyte recruitment into the lungs (Supplementary Fig. 4d-e) and increased expression of the pro-inflammatory cytokines *Il1a, Il1b* and *Tnfa* at 12 h post infection (Supplementary Fig. 4f-h).

**Fig. 2:**
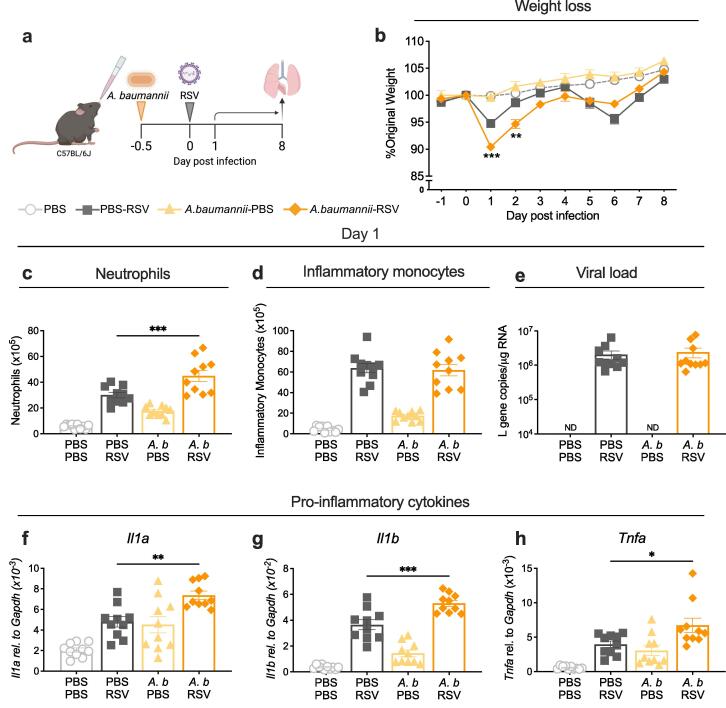
***Acinetobacter baumannii* infection before RSV infection drive more weight loss. a)** Graphic showing experimental design. Mice were infected with 5x10^5^ CFU *A. baumannii* (*A. b.*) or mock (PBS) 12 h before RSV infection (day −0.5). On day 1 after infection lungs and BAL were collected for analysis of the immune response by flow cytometry, ELISA and qPCR. **b)** Daily weight of mice represented as percentage of original body weight on day 0, in co-infected mice compared with RSV or *A. baumannii* infection only mice. **c)** Total number of neutrophils or **d)** total number of inflammatory monocytes in the lungs at day 1 after RSV infection detected using flow cytometry. **e)** Viral load represented as *L* gene copy number/µg RNA measured by qPCR and normalised to *Gapdh* on day 1 after RSV infection. ND=not detectable. Expression of **f)***Il1a*, **g)***Il1b and***h)***Tnfa* in the lung, measured by qPCR and normalised to *Gapdh*. At day 1 after infection, data are pooled from 2 experiments: n = 10. On day 8 after infection, data are pooled from 3 experiments: n = 15. Weight loss is plotted as mean ± SEM. For bar graphs, error bars represent SEM. For weight loss, a two-way ANOVA with multiple comparisons test was carried out to compare the *A. baumannii*-RSV group with RSV only controls. For all other data, a one-way ANOVA with multiple comparisons test was carried out to compare the *A. baumannii*-RSV group with RSV only controls. Asterisks represent the *p* value compared to RSV only controls; *≤0.05, **≤0.01, ***≤0.001, ****≤0.0001.

Having established the model, mice were infected with RSV 12 h after *A. baumannii* infection and weighed throughout the course of the experiment. The innate immune response was analysed at day 1 post infection (Fig. 2a). Mice infected with only *A. baumannii* did not lose any weight. Reflecting LPS pre-exposure, mice infected with *A. baumannii* before RSV rapidly lost weight within the first 2 days of RSV infection. Weight loss peaked at ∼ 10 % on day 1 post infection and was significantly increased compared to RSV only controls. At day 5 post RSV infection, mice infected with *A. baumannii* before RSV appeared to lose less weight compared to RSV only controls, but this was not statistically significant (Fig. 2b). At day 1 post infection, co-infected mice had significantly more neutrophils in the lungs but inflammatory monocytes and viral load were unchanged in co-infected mice compared to RSV only mice (Fig. 2c-e). The expression of the pro-inflammatory cytokines *Il1a, Il1b* and *Tnfa* were significantly higher in the lungs of mice infected with *A. baumannii* before RSV infection (Fig. 2f-h).

**Fig. 3:**
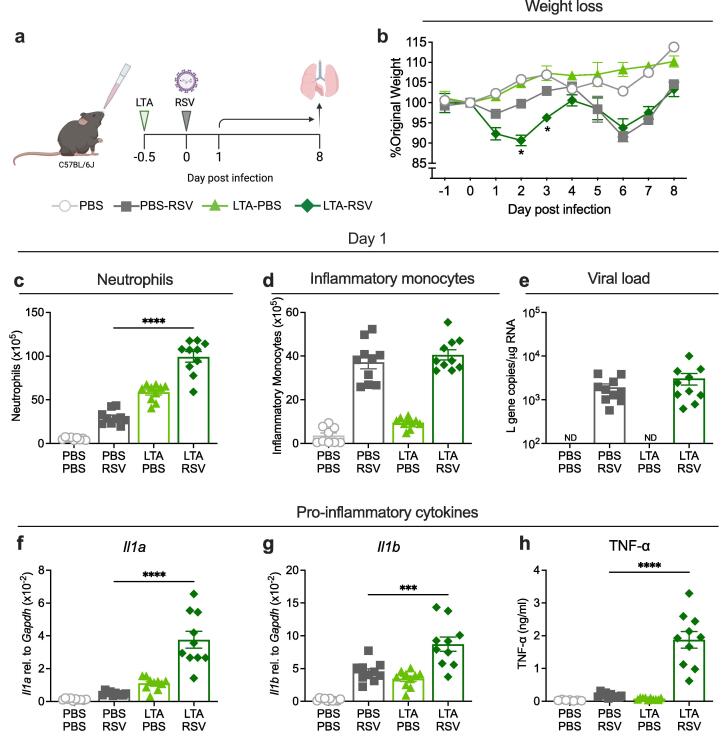
**Exposure to LTA 12 h before RSV infection increase early weight loss. a)** Graphic showing experimental design. Mice were exposed to 50 μg LTA or PBS 12 h before RSV or mock (PBS) infection (day −0.5) and weighed up to day 8 after RSV infection. On day 1 after infection, lungs and BAL were collected for analysis by flow cytometry and qPCR. **b)** Weight loss represented as percentage of original body weight on day 0. **c)** Total number of neutrophils or **d)** inflammatory monocytes in the lung at day 1 after RSV infection. **e)** Viral load represented as L gene copy number/µg RNA measured by qPCR and normalised to *Gapdh* on day 1 after RSV infection. ND=not detectable. Expression of **f)***Il1a,****g)****Il1b* in the lung measured by qPCR and normalised to *Gapdh* and **h)** TNF-α in the BAL fluid measured by ELISA. On day 1 after infection data are pooled from two experiments, mean ± SEM, n = 10. Weight loss data are from one experiment, n = 5 and plotted as the mean ± SEM. For weight loss, a two-way ANOVA with multiple comparisons test was carried out to compare the LTA-RSV group with RSV only controls. For all other data, a one-way ANOVA with multiple comparisons test was carried out to compare the LTA-RSV group with RSV only controls. Only statistically significant differences are shown. Asterisks represent the *p* value compared to RSV only controls; *≤0.05, **≤0.01, ***≤0.001, ****≤0.0001.

These findings could also be replicated by exposing mice to LTA, a PAMP associated with gram-positive bacteria, before RSV infection (Fig. 3a). Mice exposed to LTA prior to RSV experienced early weight loss and had significantly more neutrophils in the lungs but no difference in inflammatory monocytes or viral load on day 1 post infection compared to RSV infected mice (Fig. 3b-e). Expression of *Il1a, Il1b* and TNF-α were also significantly higher in the lungs of mice exposed to LTA before RSV infection compared to RSV only mice (Fig. 3f-h). This suggests that pre-exposure to either LPS, LTA or gram-negative bacteria increase the early weight loss after RSV infection.

***Early weight loss in mice exposed to LPS before RSV infection is not caused by the adaptive immune response***

We sought to identify the mechanism through which LPS pre-exposure accelerates weight loss following RSV infection. Weight loss at days 5–7 after RSV infection has been shown to be mediated by CD8^+^ T cells, but the mechanism of the earlier weight loss at day 1 post infection has not been defined.^12^ We investigated the role of T cells in disease exacerbation in this LPS-RSV model using *Rag2^-/-^* mice that lack T cells (Supplementary Fig. 5a).^56^ Despite the absence of T cells, *Rag2^-/-^* mice exposed to LPS before RSV infection lost weight similar to WT mice undergoing the same infection regime (Supplementary Fig. 5b-d). This suggests that T cells are not responsible for driving exacerbated weight loss between days 1–4 in the LPS-RSV model. The viral load at day 4 post infection was also not significantly altered in *Rag2^-/-^* compared to WT (Supplementary Fig. 5e), suggesting T cells do not reduce the viral load early during the infection in LPS exposed mice compared to RSV only mice (Fig. 1c).

***Neutrophils recruited by LPS before RSV infection do not increase early weight loss***

**Fig. 4:**
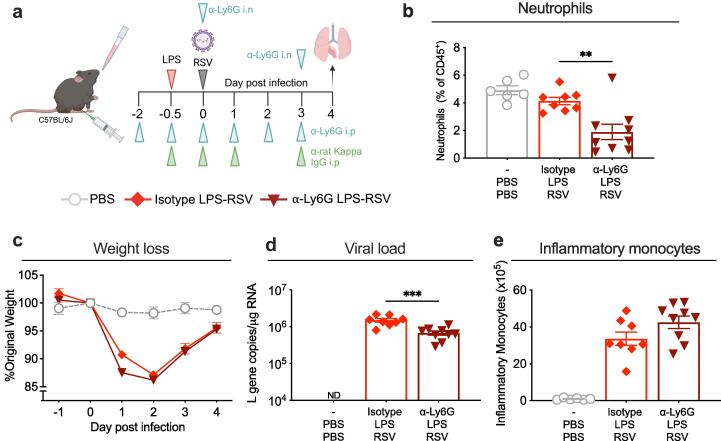
**Neutrophil depletion in mice exposed to LPS before RSV infection does not alter weight loss. a)** Graphic demonstrating neutrophil depletion protocol which was adapted from that published by Boivin et al (2020). Mice received a daily intra-peritoneal (i.p.) injection of 50 µg anti-Ly6G antibody (α-Ly6G) starting 2 days prior to RSV infection; i.p. injections of anti-rat kappa IgG (α-rat igG) on the indicated days, as well as intranasal α-Ly6G on the indicated days. Mice were intranasally exposed to LPS or PBS 12 h before RSV or mock (PBS) infection (day −0.5) and weighed up to day 4 after infection when lungs, BAL and blood were collected. **b)** Percentage of neutrophils (gated as intracellular Ly6G^+^ cells) from total CD45^+^ cells in the lungs of α-Ly6G treated mice compared to isotype controls. **c)** Daily weight of mice represented as percentage of original body weight on day 0 after infection in α-Ly6G treated mice exposed to LPS before RSV infection, compared to isotype treated mice. **d)** Viral load in the lungs represented as *L* gene copy number/µg RNA measured by qPCR and normalised to *Gapdh.* ND=not detectable. **e)** Total number of inflammatory monocytes in the lungs of α-Ly6G treated mice compared to isotype controls. Data are pooled from 2 experiments. PBS n = 6; Isotype LPS-RSV n = 8; α-Ly6G LPS-RSV n = 9. Weight loss is plotted as the mean ± SEM. For bar graphs, error bars represent SEM. For weight loss, a two-way ANOVA with multiple comparisons test was carried out to compare α-Ly6G LPS-RSV group with isotype LPS-RSV controls. For flow cytometry data and viral load data, a one-way ANOVA with multiple comparisons test was carried out to compare α-Ly6G LPS-RSV group with isotype LPS-RSV controls. Asterisks represent the *p* value compared to isotype controls; *≤0.05, **≤0.01, ***≤0.001, ****≤0.0001.

Having identified that the early weight loss associated with LPS pre-treatment prior to RSV infection was not caused by T cells, we looked for other potential candidates. Neutrophils recruited by rCXCL1 into the lungs of mice 12 h before RSV infection have been shown to drive exacerbated weight loss later during infection (day 5–7p.i.)^27^. Therefore, we investigated if neutrophils recruited by LPS before RSV infection also caused the early weight loss. Neutrophils were depleted using a combination of anti-Ly6G antibodies (α-Ly6G) and anti-rat kappa IgG antibodies (α-rat IgG) as described by Boivin et al^49^ with modifications to overcome the extensive neutrophilia driven by LPS. Mice were weighed throughout the course of the experiment up to day 4 post infection when lung, airway (BAL) and blood samples were collected (Fig. 4a). Neutrophil depletion was assessed by flow cytometry by staining for extracellular markers then subsequently permeabilised and stained for intracellular Ly6G, as extracellular epitopes could be masked by the depleting (α-Ly6G) antibodies (Supplementary Fig. 6a).^49^ Overall, the percentage of neutrophils in the lungs (Fig. 4b), airways and blood (Supplementary Fig. 6b-c) were significantly reduced compared to isotype controls at day 4 post infection. However, despite the reduction of neutrophils in the lungs of the α-Ly6G treated mice, weight loss following LPS and subsequent RSV infection, was comparable to isotype controls (Fig. 4c). Depletion of neutrophils did however result in a slight decrease in viral load at day 4 post infection (Fig. 4d). Early control of viral replication by immune cells during RSV infection has been shown to be mediated by inflammatory monocytes and not by neutrophils^10^, ^52^ and total numbers of inflammatory monocyte in the lung trended higher in the lungs following α-Ly6G depletion, but this was not significant. Therefore, it remains unclear if the significant decrease in viral load directly or indirectly is due to the loss of neutrophils or by inflammatory monocytes. Overall, these data suggest that neutrophils are not the main cell type responsible for the early weight loss during RSV infection.

***Early weight loss in mice exposed to LPS before RSV infection is partially driven by inflammatory monocytes***

**Fig. 5:**
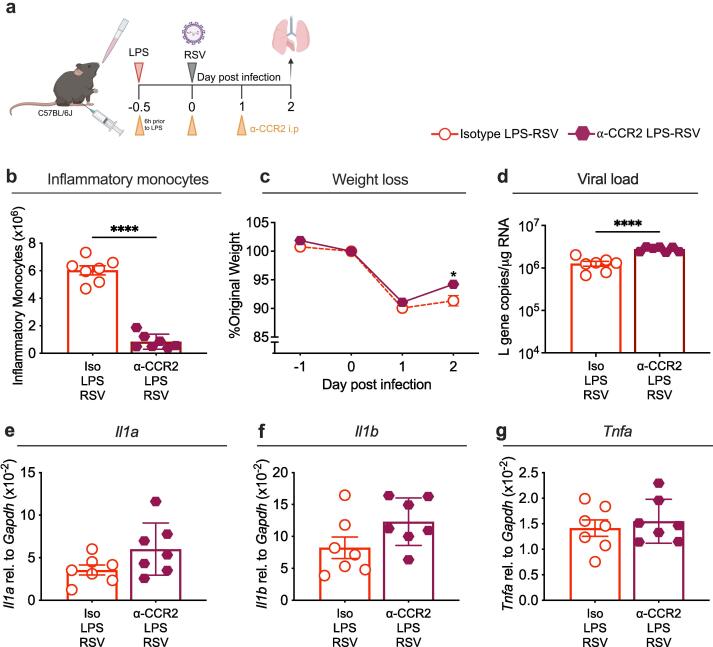
**Monocyte depletion in mice exposed to LPS before RSV infection partially ameliorates weight loss. a)** Graphic demonstrating monocyte depletion protocol. Mice received an i.p. injection of 20 µg anti-CCR2 (α-CCR2) or relevant isotype 6 h prior to intranasal LPS or PBS (day −0.5), then again on days 0 and 1. Mice were infected with RSV or mock (PBS) 12 h after LPS and weighed up to day 2 post infection. **f)** Total number of inflammatory monocytes in the lungs. **g)** Daily weight of mice represented as percentage of original body weight on day 0, in α-CCR2 treated mice compared to isotype controls. **h)** Viral load in the lungs represented as *L* gene copy number/μg RNA measured by qPCR and normalised to *Gapdh*. Expression of **i)***Il1a*, **j)***Il1b and***k)***Tnfa* in the lung, measured by qPCR and normalised to *Gapdh*. Data are pooled from 2 experiments. α-CCR2 n = 7; α-CCR2 isotype n = 7. Weight loss is plotted as the mean ± SEM. For bar graphs, error bars represent SEM. For weight loss, a two-way ANOVA with multiple comparisons test was carried out to compare α-CCR2 LPS-RSV group with isotype LPS-RSV controls. For flow cytometry, viral load and cytokine data, Student’s *t*-test was carried out to compare α-CCR2 LPS-RSV group with isotype LPS-RSV controls. Asterisks represent the *p* value compared to isotype controls; *≤0.05, **≤0.01, ***≤0.001, ****≤0.0001.

In addition to increased neutrophil recruitment, LPS exposure before RSV infection also significantly increased inflammatory monocyte recruitment into the lungs. Therefore, we investigated the contribution of inflammatory monocytes to weight loss in these mice. Monocytes can be depleted using anti-CCR2 (α-CCR2) depletion antibodies.^50^, ^57^ Mice were given 20 µg α-CCR2 or the relevant isotype by i.p. injection 6 h before intranasal LPS exposure and then again on day 0 and 1 post infection (Fig. 5a). Monocyte depletion in the lungs was confirmed using flow cytometry, with numbers being significantly reduced compared to isotype controls (Fig. 5b). Mice were infected with RSV 12 h after LPS and weighed following infection. Mice treated with α-CCR2 lost comparable weight to isotype controls on day 1 post infection. However, by day 2 post infection, α-CCR2 treated mice started to recover and had significantly less weight loss compared to isotype mice (Fig. 5c). This suggests that recruitment of monocytes plays a partial role in exacerbating weight loss in mice exposed to LPS before RSV infection. Depletion of monocytes significantly increased the viral load in these mice (Fig. 5d). As day 2 post infection is beyond the peak of the type I IFN response following RSV infection, we measured the expression of interferon stimulated genes (ISGs); *Mx1, Pkr* and *Viperin*. The expression of *Mx1* and *Viperin* were not significantly altered by monocyte depletion, whilst *Pkr* expression was significantly increased (Supplementary Fig. 8a-c). Taken together this suggests that monocytes are responsible for the significant reduction in viral load compared to RSV only mice (Fig. 1c) in line with the anti-viral effect of monocytes that we have previously shown.^11^ Monocytes have also been shown to express pro-inflammatory cytokines, in particular TNF-α, during RSV infection.^11^ Therefore, we investigated how depletion of monocytes effected the expression of *Il1a, Il1b* and *Tnfa,* which were all previously found to be elevated in mice exposed to LPS before RSV infection (Fig. 5e-g). Expression of *Il1a, Il1b* and *Tnfa* were not significantly altered in mice treated with α-CCR2 compared with isotype (Fig. 5e-g). This indicates that other cells in LPS-exposed, RSV-infected mice may be responsible for driving the elevated cytokine levels compared to RSV only mice.

***IL-1α, IL-1β and TNF-α are produced by both recruited and lung resident cells***

**Fig. 6:**
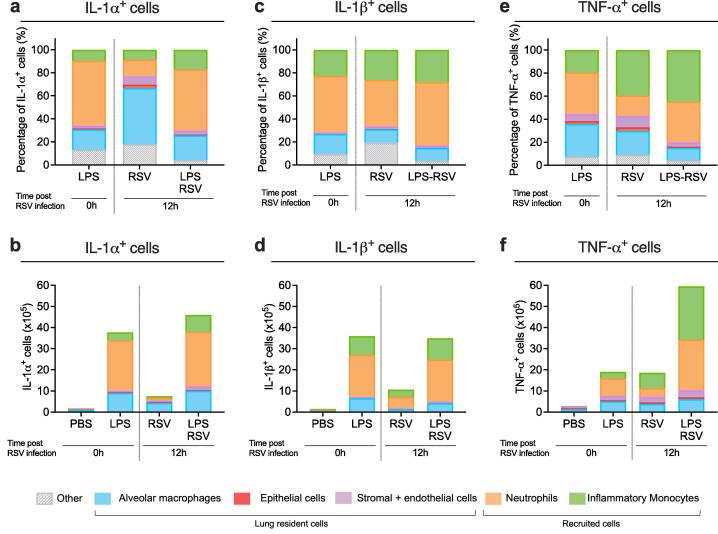
**IL-1α, IL-1β and TNF-α were produced by multiple immune cells 12 h after LPS and/or RSV infection.** Percentage contribution of lung resident AMs (blue), epithelial cells (red), and stromal + endothelial cells (pink) or recruited neutrophils (orange) and inflammatory monocytes (green) to total population of **a)** IL-1α^+^ cells, **b)** IL-1β^+^ cells or **c)** TNF-α^+^ cells, in the lung 12 h after intranasal LPS, RSV or LPS followed by RSV infection. Total number of **a)** IL-1α^+^, **b)** IL-1β^+^ or **c)** TNF-α^+^ AMs, epithelial cells, stromal + endothelial cells, neutrophils and inflammatory monocytes in the lung 12 h after intranasal LPS, RSV or LPS followed by RSV infection. Data are pooled from 2 experiments. PBS n = 6; LPS, RSV and LPS-RSV n = 8.

Increased expression of *Il1a, Il1b* and *Tnfa* in the lungs was a consistent finding in mice exposed to LPS, LTA or *A. baumannii* before RSV infection. We therefore sought to identify the cellular source of these cytokines at 12 h after LPS exposure, and 12 h after RSV infection in mice with or without pre-exposure to LPS. The total population of IL-1α^+^, IL-1β^+^ and TNF-α^+^ cells were gated from live cells (Supplementary Fig. 7). In all experimental groups, populations of AMs, neutrophils and inflammatory monocytes were identified as IL-1α^+^, IL-1β^+^ or TNF-α^+^. Of the resident lung cells, few CD45^-^ cells, including epithelial cells and cells from the stromal/endothelial compartment stained positive for any of the three cytokines (Fig. 6a-f). After RSV infection, AMs accounted for the greatest percentage of IL-1α^+^ cells. However, in mice exposed to LPS for 12 h or in mice exposed to LPS followed by RSV infection for 12 h, neutrophils accounted for the greatest percentage of IL-1α^+^ cells (Fig. 6a). Despite this, the total number of IL-1α^+^ AMs were expanded in LPS-RSV mice compared to RSV only mice (Fig. 6b). In all three conditions, neutrophils accounted for the greatest percentage of IL-1β^+^ cells, with substantial contribution from inflammatory monocytes and some from AMs (Fig, 6c). Whilst the frequency of IL-1β^+^ cells was comparable between the three groups, total numbers of IL-1β^+^ neutrophils, inflammatory monocytes and AMs were all expanded in mice exposed to LPS compared to RSV only (Fig. 6d). The greatest percentage of TNF-α^+^ cells were recruited cells but with significant contribution from AMs. Twelve hours after LPS exposure, neutrophils and AMs accounted for the greatest percentage of TNF-α^+^ cells. However, 12 h after RSV infection in mice, regardless of prior LPS exposure, inflammatory monocytes accounted for the greatest percentage of TNF-α^+^ cells (Fig. 6e). Total numbers of TNF-α^+^ neutrophils, inflammatory monocytes and AMs were all expanded in LPS-RSV mice compare to RSV only and LPS only mice (Fig. 6f). Overall, these data demonstrate that the source of IL-1α, IL-1β and TNF-α are both resident and recruited innate immune cells.

***IL-1α and TNF-α are responsible for driving early weight loss in mice exposed to LPS before RSV infection***

**Fig. 7:**
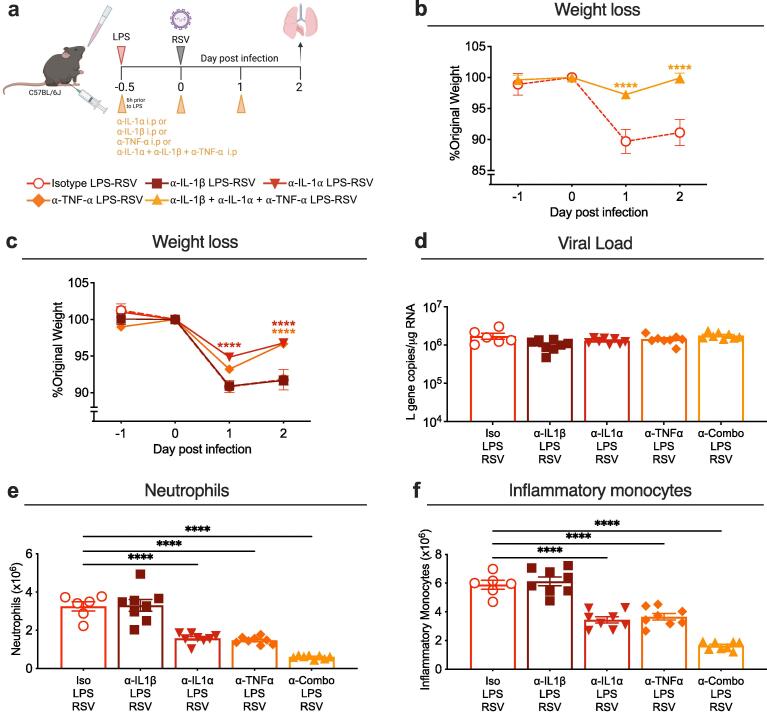
**BlockingIL-1α and TNF-αameliorates exacerbated weight loss. a)** Graphic demonstrating the cytokine depletion protocol. Mice received an i.p. injection of 250 µg anti-IL-1α (α-IL-1α), anti-IL-1β (α-IL-1β), anti-TNF-α (α-TNF-α), all three in combination (α-combo) or relevant isotype 6 h prior to intranasal LPS, then again daily. Mice were infected with RSV 12 h after LPS (day 0) and weighed up to day 2 post infection. **b)** Daily weight of mice represented as percentage of original body weight on day 0 after infection in α-IL-1α + α-IL-1β **+** α-TNF-α (α-Combo) or isotype control treated mice exposed to LPS before RSV infection. **c)** Daily weight of mice represented as percentage of original body weight on day 0 after infection in α-IL-1α, α-IL-1β, α-TNF-α or isotype control treated mice exposed to LPS before RSV infection. **d)** Viral load in the lungs of cytokine depleted mice compared to isotype controls, represented as *L* gene copy number/µg RNA measured by qPCR and normalised to *Gapdh.* Total number of **e)** neutrophils and **f)** inflammatory monocytes in the lungs. Data are pooled from 2 experiments. α-cytokine isotype LPS-RSV n = 6; α-Combo isotype LPS-RSV n = 4; α-IL-1α, α-IL-1β, α-TNF-α and α-Combo LPS-RSV n = 8. Weight loss is plotted as the mean ± SEM. For bar graphs, error bars represent SEM. For weight loss, a two-way ANOVA with multiple comparisons test was carried out to compare depletion groups with isotype LPS-RSV controls. For flow cytometry and qPCR data, a one-way ANOVA with multiple comparisons test was carried out to compare depletion with isotype LPS-RSV controls. Asterisks represent the *p* value compared to isotype controls; *≤0.05, **≤0.01, ***≤0.001, ****≤0.0001.

To determine the role of increased pro-inflammatory cytokines in the early weight loss, we used antibodies to block these cytokines. Mice were treated with anti-IL-1β (α-IL-1β), anti-IL-1α (α-IL-1α) and anti-TNF-α (α-TNF-α), alone or in combination (α-combo) or with the relevant isotype control by i.p. injection 6 h before intranasal LPS and then again on day 0 and 1. As before, mice were infected with RSV 12 h after LPS exposure and weighed up to day 2 post infection, the peak of weight loss in RSV-infected mice pre-exposed to LPS (Fig. 7a). When treated with all three blocking antibodies in combination, mice exposed to LPS prior to RSV infection lost significantly less weight on day 1 and 2 post infection (Fig. 7b). To further investigate the contribution of each of these cytokines individually to early weight loss, mice were treated with one of either α-IL-1β, α-IL-1α or α-TNF-α alone and responses compared with the relevant isotype control (Fig. 7c). Mice treated with α-IL-1β lost comparable weight to isotype-treated LPS-RSV mice (Fig. 7c). Mice treated with α-IL-1α or α-TNF-α, had partial amelioration in weight loss, which was statistically significant on days 1 and/or day 2 post infection compared to isotype controls (Fig. 7c). Cytokine blockade had no effect on viral load in the lungs compared to isotype-treated mice (Fig. 7d). This was associated with no changes to the type I IFN response measured by expression of ISGs; *Mx1, Pkr* and *Viperin* (Supplementary Fig. 8d-f). We also assessed how these early immunological changes induced by blocking IL-1α and TNF-α might affect weight loss later in the course of disease. Mice were treated with α-IL-1α and α-TNF-α in combination (α-Duo) as described previously, receiving daily i.p. injections up to day 2 post infection and weighed up to day 8 post infection. Anti-Duo treated mice were protected from weight loss on day 1–2 post infection, confirming that IL-1α and TNF-α are the main drivers of early weight loss in this model (Supplementary Fig. 8 g). However, early blockage of these cytokines had no effect on downstream weight loss, viral clearance or the T cell response at day 8 post infection (Supplementary Fig. 8 g-j). These data suggest that blocking these cytokines in combination is required to fully ameliorate early weight loss in LPS-RSV mice. with IL-1α and TNF-α playing a predominant role in driving this weight loss.

Interestingly, cytokine blockade with either α-IL-1α or α-TNF-α, but not α-IL-1β, resulted in a significant reduction in neutrophils and inflammatory monocytes in the lungs with a greater effect when the blocking antibodies were given in combination (Fig. 7d-f). Despite neutrophils and inflammatory monocytes accounting for the greatest proportion of IL-1α^+^ and TNF-α^+^ cells, these data suggest that the action of these cytokines lie upstream of monocyte and neutrophil recruitment, requiring a lung resident source. As previously identified, AMs were the primary lung resident cells that stained positive for IL-1α, IL-1β and TNF-α (Fig. 6). Out of the total AM population, over 60 % were IL-1α^+^ 12 h after RSV infection. This was increased in mice exposed to LPS for 12 h with and without subsequent RSV infection, where over 80 % AMs were IL-1α^+^ (Supplementary Fig. 9a). In contrast, 12 h after RSV infection, only 20 % AMs were IL-1β^+^ and TNF-α^+^. LPS exposure on its own did increase the percentage of IL-1β^+^ AMs to over 60 %, but this expansion was not seen in mice subsequently infected with RSV. In LPS exposed mice, with and without subsequent RSV infection, TNF-α^+^ AMs remained around 20 % of the total population (Supplementary Fig. 9a). Overall, comparing the three cytokines, IL-1α appeared to predominate the AM response with total numbers of IL-1α^+^ AMs being significantly higher than IL-1β^+^ and TNF-α^+^ AMs in LPS exposed, RSV exposed and LPS-RSV exposed mice (Supplementary Fig. 9b).

Taken together, these data suggest that in mice exposed to LPS before RSV infection, IL-1α and TNF-α work in tandem to drive increased innate inflammation, ultimately resulting in exacerbated early weight loss.

## Discussion

In this study we demonstrate that exposure to bacteria or their PAMPs before RSV infection exacerbates early disease severity in mice. This was characterised by rapid early weight loss between day 1–3 after RSV infection and was associated with prolonged elevated innate immune inflammation. Previous human challenge studies have found an increased neutrophil activation signature in the nasal mucosa before RSV infection correlated with increased incidence of symptomatic disease.^27^ We translated this into a mouse model, by recruiting neutrophils into the lungs using rCXCL1 before RSV infection which resulted in exacerbated weight loss.^27^ However, whilst LPS exposure caused significant neutrophil recruitment into the lungs, these cells were not the drivers of the increased weight loss following RSV infection in the mouse model. Instead, we have uncovered a separate mechanism linking bacterial exposure to disease following subsequent RSV infection via cytokines such as IL-1α and TNF-α.

CD8^+^ T cells have been well documented to be responsible for driving the weight loss measured at day 5–7 after RSV infection.^12^, ^58^, ^59^ However, in mice exposed to LPS before RSV infection, an increased weight loss occurred earlier at days 1–3 post infection, before CD8^+^ T cells are known to infiltrate into the airways.^60^ It was therefore no surprise that, by using *Rag2^-/-^* mice*,* which lack mature T cells, we show they play no role in driving exacerbated early weight loss in mice exposed to LPS before RSV infection.^61^

LPS binds to TLR4 resulting in an MyD88/TRIF dependent signalling cascade that cumulates in the release of multiple pro-inflammatory cytokines and chemokines.^38^ In agreement with previous literature, we show that intranasal delivery of LPS resulted in the expression of chemokines *Cxcl1* and *Ccl2,* as well as pro-inflammatory cytokines *Il1a, Il1b* and *Tnfa* and recruitment of neutrophils and monocytes.^39^, ^40^, ^62^ Therefore, intranasal LPS alters the innate immune environment in the lung before RSV is introduced, which then exaggerates the early weight loss during RSV infection. To further explore this, we infected mice with *A. baumannii* before RSV infection. *A. baumannii* is a gram-negative bacteria, thus expresses LPS.^63^ Activation of the immune response following *A. baumannii* infection is largely thought to be driven by LPS recognition by TLR4^63^ but *A. baumannii* also expresses other lipoproteins and peptidoglycans, which can be recognised by TLR2.^63^ Early weight loss was also exacerbated in mice exposed to *A. baumannii* before RSV infection, and likewise was associated with increased innate inflammation. Similar findings were also observed in mice exposed to LTA before RSV infection, suggesting the mechanism of exacerbation is not unique to gram-negative bacteria.

In addition to recruiting neutrophils, intranasal LPS also increased recruitment of inflammatory monocytes, which remained elevated in the lungs after infection compared to RSV only mice. Inflammatory monocytes have previously been shown to contribute to weight loss in mouse models of respiratory viral infection. During influenza virus infection, inflammatory monocyte depletion using α-CCR2 antibodies in juvenile mice resulted in amelioration of weight loss and improved survival.^64^, ^65^ The majority of studies focus on the weight loss that occurs between day 5 and 7 post RSV infection.^12^, ^58^, ^59^ However, RSV infection also results in some weight loss at day 1 post RSV infection.^10^ To note, *Mavs^-/-^* mice, which lack RLR signalling resulting in a lack inflammatory monocyte recruitment, do not experience this early weight loss at day 1 post RSV infection.^11^, ^66^ However, early weight loss is observed if monocytes are recruited into the lungs of *Mavs^-/-^* mice by intranasal delivery of rCCL2.^11^ When monocytes were depleted in the LPS-RSV mouse model, a partial protective effect on weight loss was observed. On day 1 post infection, α-CCR2 treated mice lost comparable weight as isotype controls, but on day 2 post infection they began to recover unlike the isotype mice. Monocytes have been shown to be an important source of TNF-α during RSV infection.^11^ Indeed, in this study we identified inflammatory monocytes as a source of TNF-α in the lung. However, monocyte depletion did not alter *Tnfa* expression in lung tissue compared to isotype controls. This is potentially due to redundancy and significant production of TNF-α from other immune cells in the LPS-RSV model.

In mice exposed to LPS, LTA or infected with *A. baumannii* before RSV infection, expression levels of the pro-inflammatory cytokines *Il1a* and *Il1b* were raised. Increased levels of TNF-α and IL-1β in nasal washes have previously been identified as biomarkers of increased disease severity following RSV infection in children.^67^ Blocking these cytokines together in mice exposed to LPS before infection with RSV, resulted in ameliorated early weight loss. Interestingly, IL-1β blockage had no effect on early weight loss, but when either IL-1α or TNF-α were depleted, early weight loss was partially ameliorated. Blocking both IL-1α and TNF-α in combination was enough to fully ameliorate weight loss suggesting these two cytokines work together to orchestrate disease in this model. Both IL-1α and TNF-α have previously been demonstrated to upregulate the expression of adhesion molecules on the surface of monocytes, promoting their recruitment and extravasation.^64^, ^65^ Indeed, blocking IL-1α, IL-1β and TNF-α in combination led to a significant reduction in the recruitment of immune cells into the lung. Therefore, it is possible that these cytokines exacerbate weight loss by orchestrating a general increase in inflammation in the lungs.

The effect of blocking IL-1α and TNF-α on inflammation suggested these cytokines lie upstream of both monocyte and neutrophil recruitment and thus require an initial lung resident source. In addition, like *Tnfa*, monocyte depletion did not significantly affect the expression of *Il1a* or *Il1b*, which further suggested redundancy or compensation in the production of these cytokines from different cell types. Both IL-1β and TNF-α have previously been shown to be released by AMs in response to RSV^68^, ^69^. Viral infections have also been shown to stimulate the release of IL-1α from epithelial cells.^70^ however, very few of the recovered epithelial cells or cells from the stromal/endothelial compartment stained positive for IL-1α, IL-1β or TNF-α following LPS exposure or LPS followed by RSV infection. Instead, AMs were identified as the predominant lung resident cells to produce these three cytokines.

Recruited neutrophils and inflammatory monocytes 12 h after LPS or LPS followed by RSV infection, accounted for greater proportions of all IL-1α^+^, IL-1β^+^ and TNF-α^+^ cells compared to lung resident cells. This may be indicative of positive feedback generated by IL-1α, IL-1β and TNF-α receptor signalling, which drive the recruitment of more inflammatory cells that further potentiate the release of these cytokines.^71^, ^72^ The significant contribution of neutrophils, inflammatory monocytes and AMs to IL-1α and TNF-α production may explain why depleting only one of these cell populations does not protect mice from early weight loss.

Blocking TNF-α in mouse models has previously been shown to ameliorate the early weight loss measured at day 1 post RSV infection.^73^ Similarly, prolonged release of TNF-α by CD8^+^ T cells at days 5–7 post RSV infection in mice has also been associated with increased weight loss at this time point.^74^ In these models, it has been suggested that TNF-α results in weight loss by directly acting on neurons that exert control over appetite during the sickness response,^73^ this has also been observed with IL-1α.^75^ When injected into the cerebral ventricles of mice, TNF-α or IL-1β can drive loss of appetite which results in rapid weight loss^76^. However, unlike TNF-α and IL-1α, IL-1β blockage has previously been shown to exacerbate weight loss on day 7 post RSV infection, suggesting it may play a more protective role.^75^ It is unclear if the prolonged expression of these cytokines in the lung will subsequently lead to elevated levels in the circulation and brain. However, circulating TNF-α, IL-1α and IL-1β have all been shown to cross the blood brain barrier in mice.^77^, ^78^, ^79^ It is possible that in addition to driving dysregulated inflammation, these cytokines can directly act on appetite to exacerbate weight loss.

Given that both IL-1α and IL-1β signal through the same receptor IL-1R, it was surprising that only IL-1α blockade influenced disease outcomes. It has previously been reported that IL-1β blockade using a similar protocol in RSV mouse models can affect weight loss later in the course of infection.^75^ This is not the first report of IL-1α and IL-1β seemingly having differential roles during infection. In a mouse model of sub-cutaneous *Streptococcus* infection, IL-1α was shown to induce unique transcriptomic changes to the liver, whilst IL-1β had more effect on the spleen transcriptome.^80^ IL-1α and not IL-1β has also been shown to drive neutrophil recruitment to the lungs during *Legionella pneumophila* infection in mice.^81^ The differential effects of blocking IL-1α or IL-1β in the LPS-RSV model is therefore likely due to spatial and temporal differences in the production and signalling of these cytokines. The identification of AMs as the lung resident source of both IL-1α and IL-1β may support this theory, as IL-1α^+^ AMs greatly outweighed IL-1β^+^ AMs 12 h after LPS and subsequent RSV infection. This may suggest IL-1α is more rapidly produced and released from AMs than IL-1β, or even pre-formed. Supporting this, whilst both *Il1a* and *Il1b* gene expression were increased in LPS-RSV mice on day 1 post infection, only IL-1α and not IL-1β protein was detectable in the airways. The release of IL-1α by macrophages into the alveolar space has previously been shown to occur more rapidly than IL-1β in response to inhaled silica.^72^ This was found to be due to release of constitutive stored IL-1α in AMs, which was a requirement to precede release of IL-1β from AMs.^72^ Furthermore, both IL-1α and IL-1β are released in a pro-form, which require cleavage by enzymes. Whilst pro-IL-1β requires cleavage by caspase 1 to become functionally active, pro-IL-1α can signal via IL-1R and cleavage by calpain or granzyme B only further potentiates its activity.^82^, ^83^ Therefore, this suggests that IL-1α can act earlier than IL-1β following its release from AMs in the lung in response to LPS.

Overall, these findings demonstrate exposure to gram-negative bacteria or LPS before RSV infection exacerbated disease early during the innate phase of the inflammatory response. Consistent with this, the innate immune response in the lungs was significantly elevated for a prolonged duration. Ultimately, we propose this loss in regulation of the innate response is orchestrated by elevated expression of IL-1α and TNF-α. The consistent findings across models of LPS, LTA and *A. baumannii* exposure before RSV infection suggests this mechanism is not pathogen specific nor unique to gram-negative bacteria. It is possible that humans exposed to bacteria or bacterial PAMPs in the environment or have an altered microbiota before RSV infection, may also exhibit rapid onset of symptoms and deterioration in health associated with uncontrolled and prolonged innate inflammation. It is also possible that this connects to increased disease severity after respiratory viral infections in people with heightened inflammation such as inflamaging or obesity, where cytokines such as IL-1α and TNF-α could be increased in the mucosa before infection. Therefore, this mouse model may help us better understand the drivers of disease severity in patients co-infected with bacteria and RSV.

## Materials and Methods

**Mice**

All *in vivo* experiments were carried out using 7–10 weeks old, male and female C57BL6/J mice purchased from Charles River UK. *Rag2^-/-^* mice (provided by Marina Botto, Imperial College London) were bred in-house in specific pathogen-free conditions. Animal experiments were reviewed and approved by the Animal Welfare and Ethical Review Board (AWERB) within Imperial College London and approved by the UK Home Office in accordance with the Animals (Scientific Procedures) Act 1986.

**Intranasal** **bacterial antigen exposure**

Mice were sedated with isoflurane gas and received an intranasal dose of either 1 µg lipopolysaccharide (LPS) (Invivogen, US), 50 µg lipoteichoic acid (LTA) (Invivogen) diluted in 100 µl PBS. Intranasal PBS administration was used as a control in all experiments.

**Bacterial culture and infection**

*Acinetobacter baumannii* strain ATCC 17978 was streaked onto an LB agar plate from frozen glycerol stock, and incubated at 37 °C overnight, up to 1 week before infections as described previously.^48^ A sweep of dense colony growth was taken from the LB agar plate using an inoculating loop and dipped into 5 ml LB broth. The inoculated broth was incubated at 37 °C, 250 rpm overnight to generate a liquid culture. For infections, 200 μl of overnight liquid culture was added to 20 ml LB broth in a 250 ml Erlenmeyer flask and grown at 37 °C, 250 rpm for 2.5–3 h until the culture had reached an OD_600_ of 0.7. The culture was then pelleted, washed with PBS and resuspend in PBS. At this point, the culture was assumed to be approximately 1x10^10^ CFU/ml and was diluted 1:2,000 to generate ∼ 5x10^6^ CFU/ml infection culture. Mice were sedated with isoflurane gas and infected with 100 μl of the infection culture. Thus, each mouse mice received ∼ 5x10^5^ CFU. To calculate the true CFU used to infect the mice, the infection dose was serially diluted 1 in 10 and 6 dilutions (10^-1^-10^-6^) were dripped onto LB agar plates in parallel strips using a multichannel. The plates were incubated at 37 °C overnight and CFU/ml was calculated by counting the colonies. In these experiments the final dose was 5-8x10^5^ CFU.

**Viral infection**

For RSV infections, RSV (plaque purified human RSV, originally A2 strain from ATCC, US) was grown in HEp2 cells cultured in antibiotic free, DMEM+2% FCS. Mice were sedated using isoflurane gas and subsequently infected with 7x10^6^ focus-forming units (FFU) in 100 µl. Mice were infected with RSV 12 h after receiving an intranasal dose of LPS, LTA, *A. baumannii* or PBS. Weight loss was monitored as a measure of disease severity.

**Neutrophil depletion**

The protocol for neutrophil depletion was based on that previously published by Boivin et al^49^ and modified to overcome extensive neutrophilia generated by LPS exposure. Mice received a daily intraperitoneal (i.p.) injection of 50 μg anti-Ly6G antibody (α-Ly6G; 1A8; Assay Genie, IE) or isotype control diluted in 100 µl PBS, starting 2 days before RSV infection (1 day before LPS). Mice then received an i.p. injection of 50 µg anti-rat kappa IgG (α-rat IgG; MAR 18.5; Assay Genie) diluted in 100 µl PBS, 1 day before RSV infection (same day as LPS) and then again on days 0, 2 and 3 after RSV infection. On day 0, RSV and 25 µg α-Ly6G were diluted together in 100 µl PBS and delivered intranasally as described above. Mice also received 50 µg α-Ly6G intranasally on day 3 after infection. On day 4 after infection mice were culled and depletion was assessed in the lung, BAL and blood by flow cytometry as described below.

**Cytokine depletion**

Mice received an i.p. injection of 250 µg anti-IL-1α (Assay Genie), anti-IL-1β (Assay Genie), anti-TNF-α (Assay Genie) or all three in combination diluted in 200 µl PBS, starting 6 h before intranasal LPS exposure and then again once daily up to day 2 post infection. Isotype control mice were given either 250 µg or 750 µg Armenian Hamster IgG.

**Monocyte depletion**

Monocyte depletion was carried out using the anti-CCR2 antibody MC21.^50^ Mice were injected i.p. with 20 µg anti-CCR2 or an IgG2b isotype-matched control (Assay Genie) diluted in 100 µl PBS, 6 h before intranasal LPS and then again daily throughout the experiment.

**Bronchoalveolar lavage (BAL) and airway cell processing**

Mice were culled at either day 1, 2, 4 or 8 after infection by intra-peritoneal injection of phenobarbital (Pentoject, Animalcare, UK). To collect airway cells, the bronchoalveolar lavage (BAL) was obtained by flushing the lungs three times using 1 ml PBS with 0.5 mM EDTA, via a small incision in the trachea. Following centrifugation, the BAL supernatants were reserved and stored at −80 °C for ELISA. Airway cells were resuspended in 0.15 M NH_4_Cl, 1.0 mM KHCO_3_, 0.1 mM EDTA (ACK lysis buffer), to lyse any red blood cells. Total live airway cells were quantified using 0.1 % Trypan Blue (Thermo Fisher Scientific, US) dead cell exclusion.

**Lung cell processing**

After BAL was collected and before excision, lungs were perfused with PBS by intracardiac injection. The upper right lobe was reserved, snap frozen in liquid nitrogen then stored at −80 °C for later use for RNA extraction. For isolation of CD45^+^ cells only, the remaining 4 lobes were suspended in DMEM+10 % FCS, 2 mM L-glutamine, 100U/ml penicillin, 10 μg/ml streptomycin (cDMEM) + 1 mg/ml collagenase D and 30 μg/ml DNase I in MACS C-tubes (Miltenyi Biotec, DE). Mechanical homogenisation was carried out using the gentleMACS dissociator (Miltenyi Biotec) according to manufacturer’s instruction. Samples were incubated at 37 °C for 1 h. The suspensions were then homogenised once again using the gentleMACS dissociator. For isolation of CD45^+^ and CD45^-^ cells for intracellular cytokine staining, the lung was digested in 5 mg/ml Liberase + 30 μg/ml DNase + 5 ng/ml Brefeldin A (BFA) for 40 mins at 37 °C. Digested lung was then homogenised by mashing through a 100 μm cell strainer. Red blood cell lysis was carried out as described above using ACK lysis buffer or for isolation of CD45^+^ and CD45^-^ cells using Miltenyi Red Blood Cell Lysis buffer. Samples were resuspended in PBS before being filtered through a 100 μm cell strainer generating a single cell suspension. For cytokine staining, cells were resuspended in cDMEM+5ng/ml BFA. Total live lung cells could then be quantified using 0.1 % Trypan Blue exclusion to count the cells per sample then multiplying by 1.2 to account for the lobe reserved for RNA extraction.

**Histological assessment**

Mice were exposed to LPS or PBS and 12 h later infected i.n. with RSV or mock infected. A day later, mice were sacrificed by a fatal dose of pentobarbital injected i.p.. Lungs were inflated with 1.5 ml of PBS, fixed in 10 % Formalin (Sigma-Aldrich) for 16 h and embedded in paraffin blocks. Three sections of 4 mm at least 150 mm apart were stained for H&E according to standard procedures. Sections were scanned using the Axio Scan Z1 slide scanner (Zeiss, Germany) and analysed using the Zen Blue software (Zeiss, Germany).

**Blood cell processing**

Blood was collected post-mortem from the femoral artery and immediately suspended in PBS+1mM EDTA to prevent clotting. Red blood cell lysis was carried out using ACK buffer and samples were resuspended in 200 µl FACs buffer (PBS+5% BSA+0.1 mM EDTA) for flow cytometry staining.

**Flow cytometry**

Before antibody staining, 2.5x10^6^ lung cells, all BAL cells and all blood cells, were suspended in purified rat IgG2b anti–mouse CD16/CD32 receptor antibody (BD Bioscience, US) diluted 1:200 in PBS+5% BSA+1mM EDTA (FACS buffer) to prevent non-specific Fc receptor binding. The cells were then stained with CD45 (30-f11), Siglec-F (E50-2440), Ly6G (1A8), CD11c (HL3), CD64 (X54-5/7.1), CD11b (M1/70), CD19 (1D3), CD3 (17A2 or 145-2C11), CD4 (GK1.5), CD8 (53–6.7), CD69 (H1.2F3), CD49b (HMa2) and live/dead Aqua dye (Invitrogen, US) diluted in PBS, for 30 mins at 4 °C. Following staining, cells were fixed using Cytofix™ Fixation Buffer (BD) for 20 mins at 4 °C, then resuspended in FACS buffer. For intracellular staining of Ly6G, fixed cells were resuspended in permeabilisation buffer for 20 min at 4 °C. Subsequently cells were stained with Ly6G in a separate fluorophore used for extracellular staining, diluted in permeabilisation buffer for 30 mins at 4 °C. Cells were then washed and resuspended in FACS buffer and immediately taken for acquisition using a BD LSR Fortessa. For intracellular staining of cytokines, lung cells were incubated in cDMEM+GolgiStop (BD) for 1 h at 37 °C. Cells were then treated with FC block and stained for extracellular markers as described above but in FACS buffer or PBS containing BFA. Cells were fixed and permeabilised as described above for intracellular staining and stained for IL-1α, IL-1β and TNF-α. Cells were then washed and resuspended in FACS buffer and immediately taken for acquisition using a BD LSR Fortessa. Before acquiring samples, the laser channels were compensated using compensation beads. Samples were acquired with a stopping gate of 2.5x10^5^ single, live CD45^+^ cells. Data analysis was carried out using FlowJo (BD).

**E** **LISA** **s**

Quantification of IFN-α and IL-6 were carried out by ELISA as previously described.^51^ Quantification of TNF-α was carried out using R&D DuoSet ELISA kit following the manufacturer’s protocol. Absorbance was measured at 450 nm using FLUOstar Omega (BMG Labtech, UK) plate reader and analysed using the Mars software (BMG Labtech).

**Multiplex Assay**

A flow cytometry based multiplex panel was designed by Assay Genie to detect IL-1α and IL-1β and run following the manufacturer’s protocol. Briefly, beads with differing fluorescent intensity were pre-coated with antibodies specific to IL-1α or IL-1β. These beads were incubated for 1hr with standards and BAL supernatants at room temperature. Plates were washed using a vacuum filter plate washer then beads were incubated with biotinylated antibodies specific for IL-1α or IL-1β for 30 mins at room temperature. Following washing, beads were then incubated with streptavidin-PE for 20 mins at room temperature. Subsequently, beads were acquired by flow cytometry and PE intensity was used to extrapolate cytokine concentrations from the standard curve. Data analysis was carried out by Assay Genie.

**RNA extraction and quantitative RT-PCR**

The upper right lung lobe was homogenised using a TissueLyser LT (Qiagen, DE) and RNA extraction was carried out using the RNeasy Mini kit (Qiagen) following the kit protocol. An on column 15 min DNase (Qiagen) digest step was included. Eluted RNA was quantified using a NanoDrop (Thermo Fisher Scientific) and 2 μg of RNA were converted to cDNA using High Capacity RNA-to-cDNA kit (Applied Biosystems, US). All qPCRs were carried out using Quantitect Probe PCR Master Mix (Qiagen). For *Il1a, Il1b, Mx1* and *Gapdh,* Taqman (Thermo Fisher Scientific) gene expression assay primers and probes were used. For *Ifnb, Tnfa, Pkr, Viperin* and L gene custom primers and probes were used as previously described.^52^, ^53^, ^54^ qPCRs were run on a Taqman 7500 fast PCR machine. CT values were obtained, averaged and normalised to *Gapdh.* The scale was inverted by raising 2 to the power of −CT (normalised to *Gapdh).* For L gene, copy number was calculated by the Taqman 7500 fast PCR machine software from a standard curve.

**Bacterial load**

Lungs were excised and placed into antibiotic free DMEM then homogenised through a 100 μm cell strainer. The resulting homogenised lung, as well as BAL fluid were serially diluted 1:10 and dripped onto LB agar plates using a multichannel. Plates were incubated overnight at 37 °C to allow colony growth. Colonies were then counted at appropriate dilutions and used to calculate CFU/ml.

**Statistical analysis**

All data were plotted and statistically analysed using Prism v9 software (Graphpad). Data are presented as mean ± SEM. Normal distribution was assessed using Q-Q plots and Shapiro-Wilk test. As time course data were not paired, one-way ANOVA with Tukey’s after hoc test was run to compare between the multiple groups at each time point. In weight loss curves where data were paired, two-way ANOVA was run. Data were considered statistically significant if *p* ≤ 0.05.

**Author contribution**

AO designed, performed, and analysed the experiments. AF designed and performed specific experiments and reviewed the paper. AML and ZW performed specific experiments and reviewed the paper. MM provided α-CCR2 blocking antibodies and reviewed the paper. SH and JT provided the *A. baumannii* strain, gave advice and reviewed the paper. CJ supervised the project and designed the experiments. AO and CJ wrote the paper.

**Disclosure and competing interest statement**.

The authors declare no competing interests.

## CRediT authorship contribution statement

**Amber R. Owen:** Writing – review & editing, Writing – original draft, Visualization, Investigation, Formal analysis, Data curation, Conceptualization. **Ana Farias:** Writing – review & editing, Investigation. **Anne-Marie Levins:** Writing – review & editing, Investigation. **Ziyin Wang:** Investigation, Methodology. **Sophie L. Higham:** Writing – review & editing, Methodology. **Matthias Mack:** Writing – review & editing, Methodology. **John S. Tregoning:** Writing – review & editing, Methodology. **Cecilia Johansson:** Writing – original draft, Supervision, Project administration, Funding acquisition, Conceptualization.

## Declaration of competing interest

The authors declare that they have no known competing financial interests or personal relationships that could have appeared to influence the work reported in this paper.
