# Supplementary material for: Exposure to bacterial PAMPs before RSV infection exacerbates innate inflammation and disease via IL-1α and TNF-α
Source: Mucosal Immunol. 2024 Dec;17(6):1184–98. doi: 10.1016/j.mucimm.2024.08.002 (PMC11631774; doi:10.1016/j.mucimm.2024.08.002)
Supplement: Supplementary Data 1 [file mmc1.pdf]

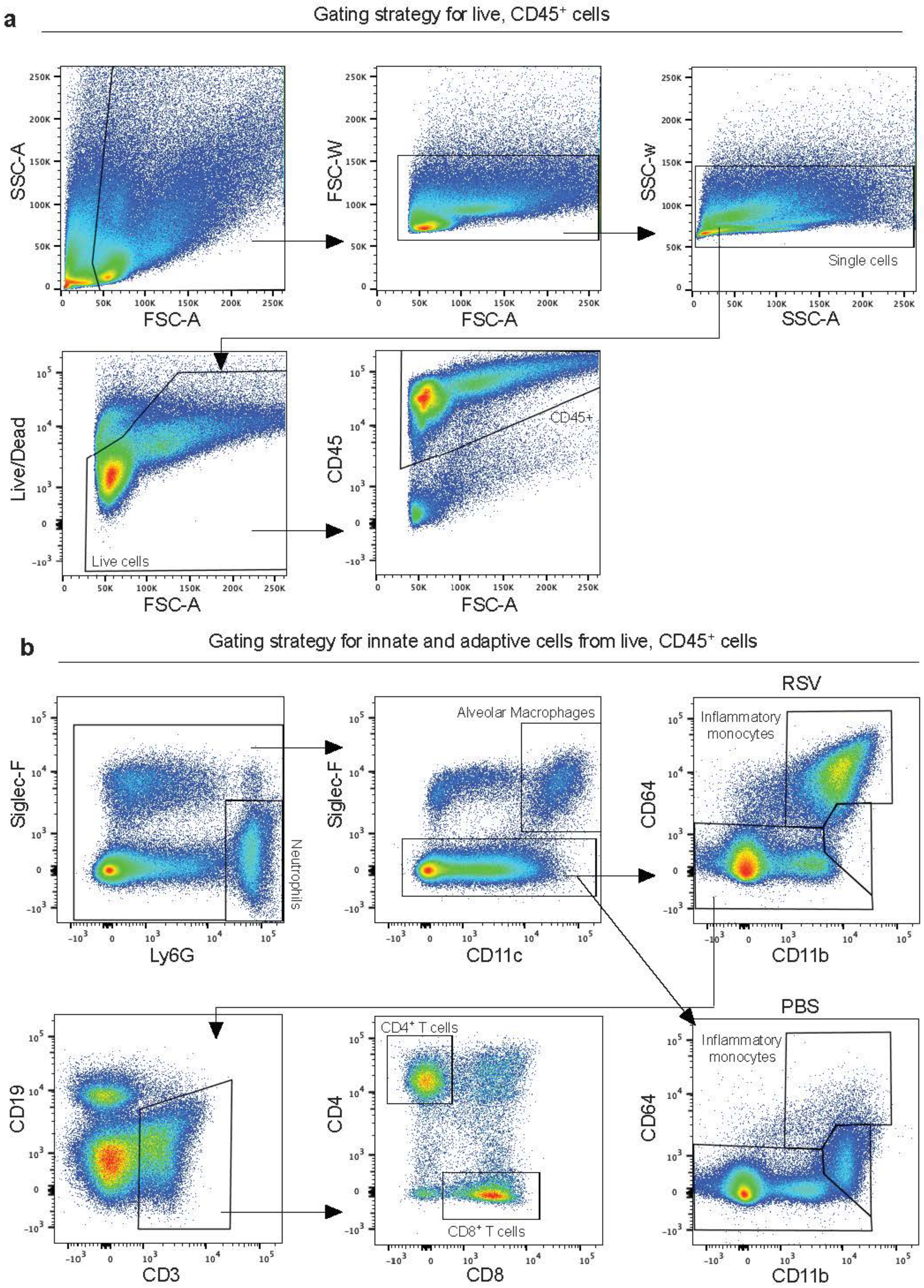
a
Gating strategy for live, CD45+ cells
SSC-A
FSC-A
FSC-W
FSC-A
SSC-w
SSC-A
Single cells
Live/Dead
FSC-A
Live cells
CD45
FSC-A
CD45+
b
Gating strategy for innate and adaptive cells from live, CD45+ cells
Siglec-F
Ly6G
Neutrophils
Siglec-F
CD11c
Alveolar Macrophages
RSV
CD64
CD11b
Inflammatory monocytes
CD19
CD3
CD4
CD8
CD4+ T cells
CD8+ T cells
PBS
CD64
CD11b
Inflammatory monocytes

Supplementary Figure 1. Immune cell gating strategy. a) Example flow cytometry plots showing lung cells from an RSV infected mouse demonstrating the gating strategy for live, $CD45^+$ cells. **b)** Example flow cytometry plots showing lung cells from an RSV infected mouse demonstrating the gating strategy for innate and adaptive immune cell populations including neutrophils, alveolar macrophages, inflammatory monocytes, $CD4^+$ T cells and $CD8^+$ T cells. An example plot for the inflammatory monocyte gating from a PBS is mice is also shown as indicated. All flow gating was carried out using FlowJo software.

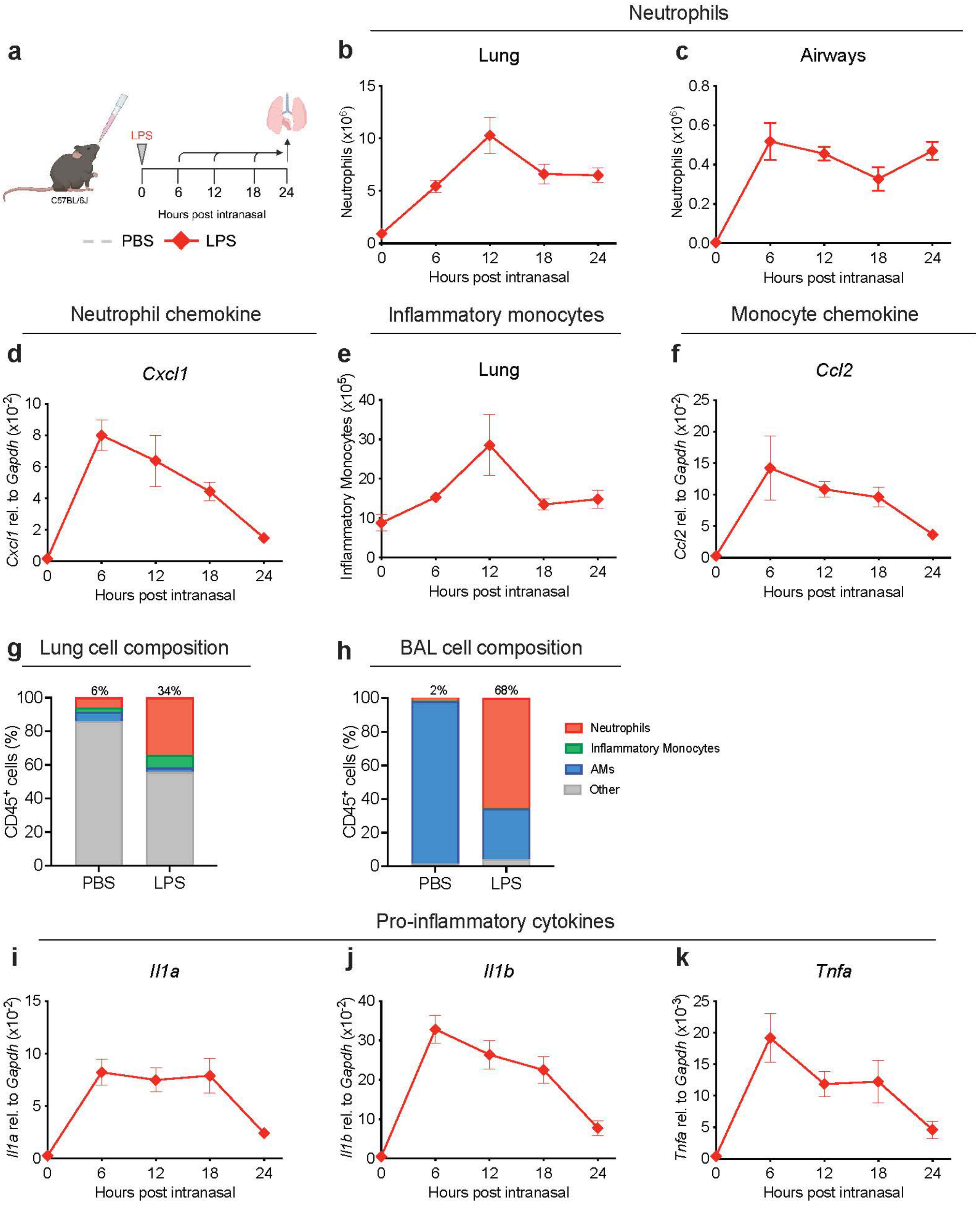

Supplementary Figure 2. Immune responses following intranasal LPS. a) Graphic describing the experimental setup. Mice were intranasally exposed to either 1μg LPS or PBS as a control. 6, 12, 18 or 24h later, airway (BAL) and lung cells were collected for analysis by flow cytometry, ELISA and qPCR. Total number of neutrophils in the **b)** lung and **c)** airways (BAL) following LPS (red diamonds) compared to PBS baseline (grey dashed line). **d)** Expression of *Cxcl1* in the lung,

measured by qPCR and normalised to *Gapdh.* **e)** Total numbers of inflammatory monocytes in the lungs following LPS. **f)** Expression of *Ccl2* in the lung, measured by qPCR and normalised to *Gapdh.* Frequency of cells in the lung **g)** and BAL **h)** 12h post PBS or LPS administration. Expression of **i)** *Il1a,* **j)** *Il1b* and **k)** *Tnfa* in the lung, measured by qPCR and normalised to *Gapdh*. Data for 12h after exposure to LPS or PBS baseline are shown as day 0 data in Fig. 1. 0h represent the mock (PBS) infected mice pooled from different time points. Data are pooled from two independent experiments. LPS n=6; PBS baseline n=10. LPS data are plotted as mean ± SEM.

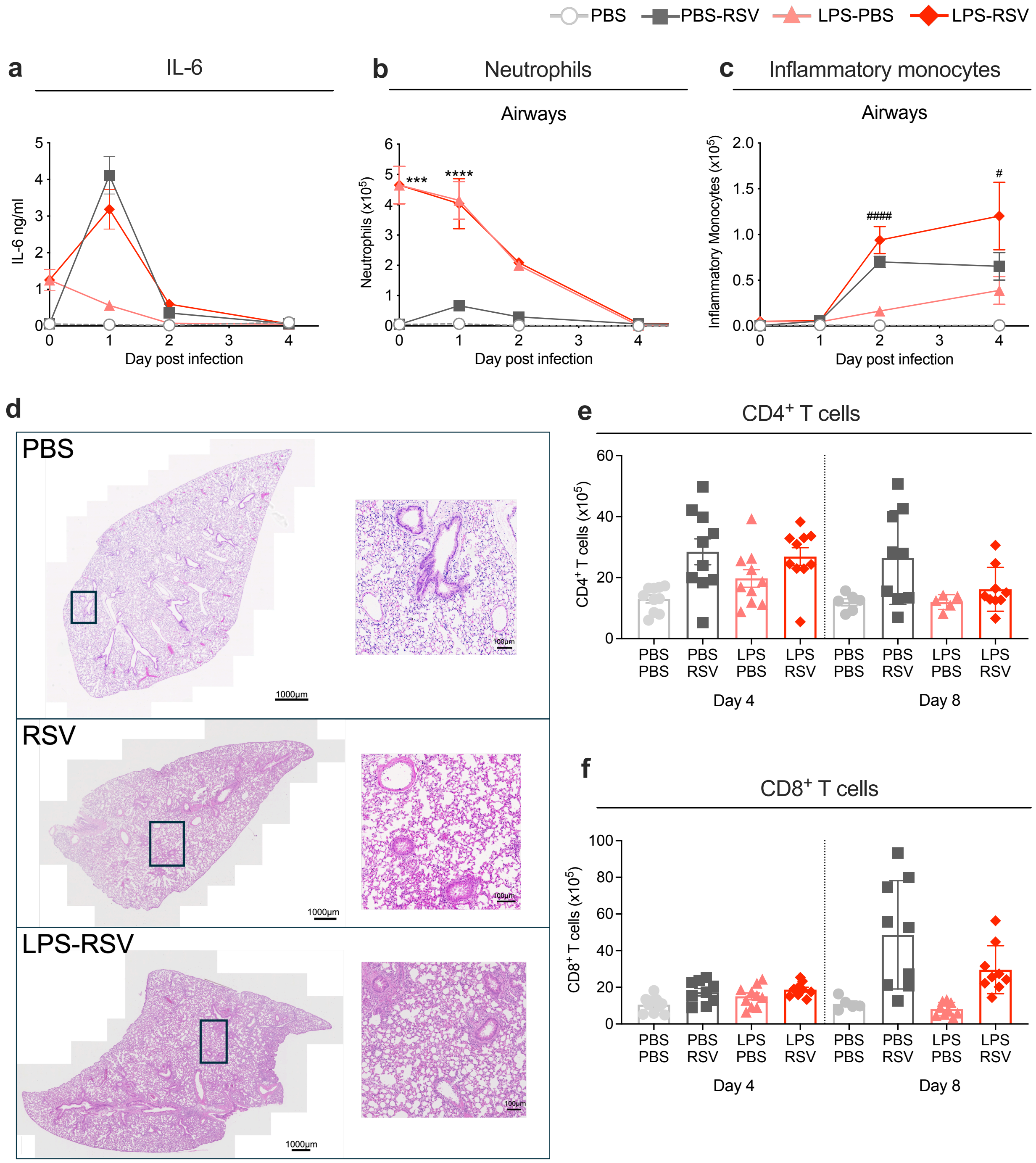

Supplementary Figure 3. Innate and adaptive immune responses in the airways and lungs of mice exposed to LPS before RSV infection. a) Levels of IL-6 in the BAL supernatant measured by ELISA. **b)** Total number of neutrophils or **c)** inflammatory monocytes in the airways over time following RSV infection detected by flow cytometry. **d)** C57BL/6J mice were exposed to LPS or vehicle control (PBS) i.n., followed by RSV infection 12h later. Lungs were collected 24h after RSV infection and processed for H&E staining. Representative sections from a control mouse (PBS), an infected only mouse (RSV) and a mouse exposed to LPS prior to RSV infection (LPS-RSV) are shown. Scale bars correspond to 1000µm or 100µm as indicated. **e)** CD4$^+$ T cells and **f)** CD8$^+$ T cells in the lungs at days 4 and 8 after RSV infection identified using flow cytometry. At days 0-4 data are

pooled from 2 experiments per time point. Day 0 data are the same as presented in Fig.1 for 12h after exposure to LPS or PBS baseline. At day 1, 2 and 4 after infection, n=10. At day 8 PBS only and LPS only, data are from 1 experiment n=5; RSV only and LPS-RSV, data are pooled from 2 experiments n=9. Time courses are plotted as mean ± SEM. For bar graphs, error bars represent SEM. A one-way ANOVA with multiple comparisons test was carried out for each timepoint. Asterisks represent the *p* value for LPS-RSV data compared to RSV infected controls; *≤0.05, **≤0.01, ***≤0.001, ****≤0.0001. Hashtags represent the *p* value for LPS-RSV compared to LPS only controls; #≤0.05, ##≤0.01, ###≤0.001, ####≤0.0001.

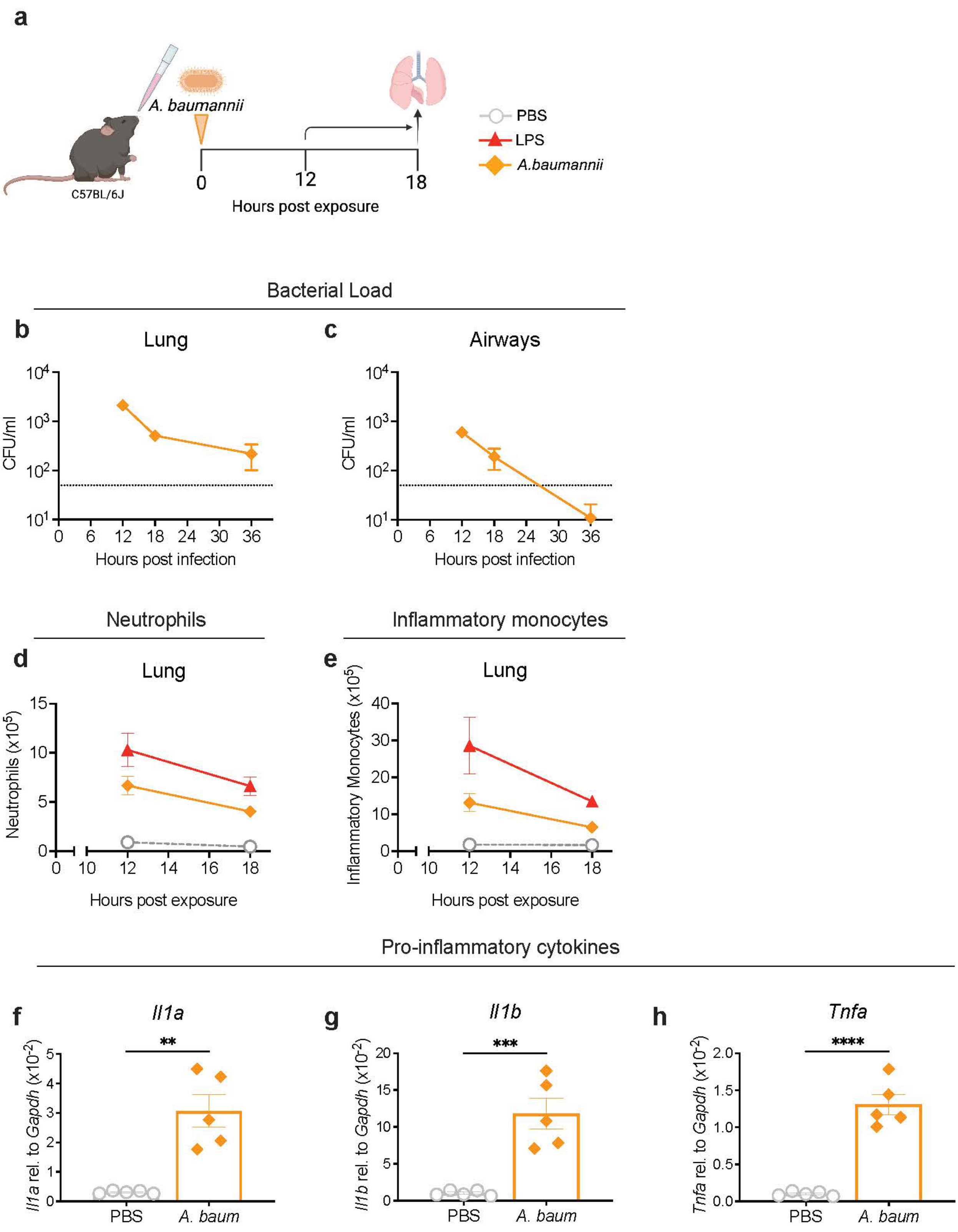

Supplementary Figure 4. Immune response following intranasal *Acinetobacter baumannii* infection. a) Graphic showing experimental design. Mice were intranasally administered 5-8x10^5 CFU *Acinetobacter baumannii.* At different time points, lungs and BAL were collected and processed

either for CFU counts of for flow cytometry and qPCR. **b)** CFU/ml counted from lung homogenate plated onto LB agar at 12, 18 or 36h after *A. baumannii* infection. **c)** CFU/ml counted from BAL plated onto LB agar at 12,18 or 36h. Dotted line represents the limit of detection determined from samples collected from PBS control mice. **d)** Total numbers of neutrophils in the lungs and airways following intranasal *A. baumanni* compared to LPS at 12 and 18h. **e)** Total numbers of inflammatory monocytes in the lungs following intranasal *A. baumanni* compared to LPS. Expression of **f)** *Il1a,* **g)** *Il1b* and **h)** *Tnfa* in the lung at 12h after *A. baumannii* infection, measured by qPCR and normalised to *Gapdh*. Data are from one experiment per time point, n=5. LPS data are the same data originally presented in Figure 1. An unpaired student's *t*-test was carried out comparing *A. baumanni* infected mice to PBS controls. Asterisks represent the *p* value; *≤0.05, **≤0.01, ***≤0.001, ****≤0.0001.

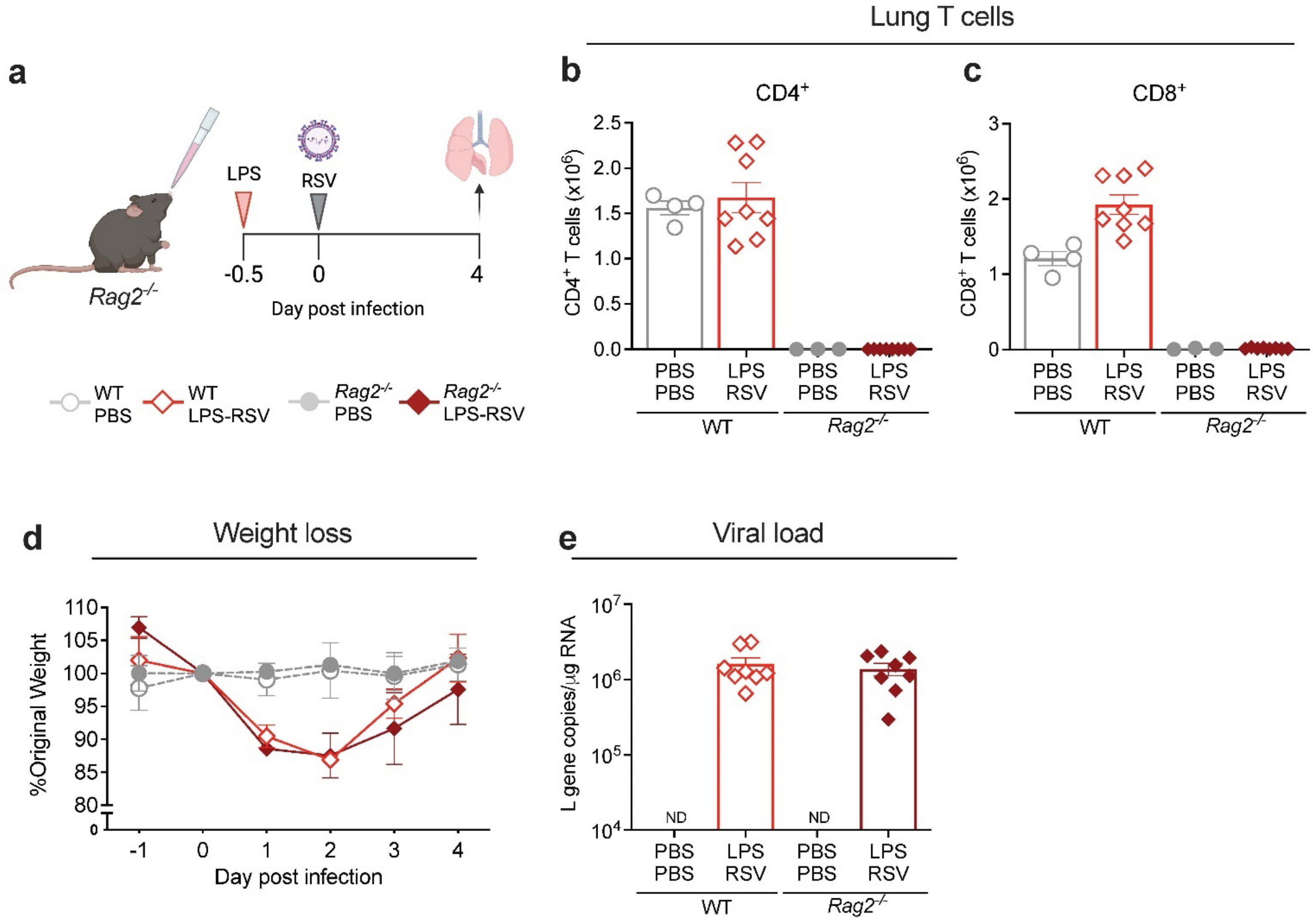

Supplementary Figure 5. Exposure to LPS and subsequent RSV infection in *Rag2*$^{-/-}$ mice are not different to WT mice. a) Graphic showing experimental design. *Rag2*$^{-/-}$ and C57BL/6J (WT) mice were intranasally exposed to LPS 12h before RSV infection. Mice were weighed up to day 4 at which point lungs and BAL were collected for flow cytometry analysis. **b)** Total number of CD4^{+} T cells or **c)** total number of CD8^{+} T cells in the lung at day 4 after infection in *Rag2*$^{-/-}$ compared to WT mice. **d)** Daily weight of mice represented as percentage of original body weight on day 0. **e)** Viral load in the lungs measured as L gene copy number/μg RNA measured by qPCR and normalised to *Gapdh*. ND= not detectable. Data are pooled from 2 experiments. WT PBS only n= 4; WT LTA- RSV n=8; *Rag2*$^{-/-}$ PBS n=3; *Rag2*$^{-/-}$LPS-RSV n=8. Weight loss is plotted as the mean ± SEM. For bar graphs, error bars represent SEM. For weight loss, as data were paired, a two-way ANOVA with multiple comparisons test was carried out to compare the *Rag2*$^{-/-}$ LPS-RSV group with the WT LPS-RSV group. No statistical significance was found. For flow cytometry data, a one-way ANOVA with multiple comparisons test was carried out to compare *Rag2*$^{-/-}$ mice with WT controls. Asterisks represent the *p* value; *≤0.05, **≤0.01, ***≤0.001, ****≤0.0001.

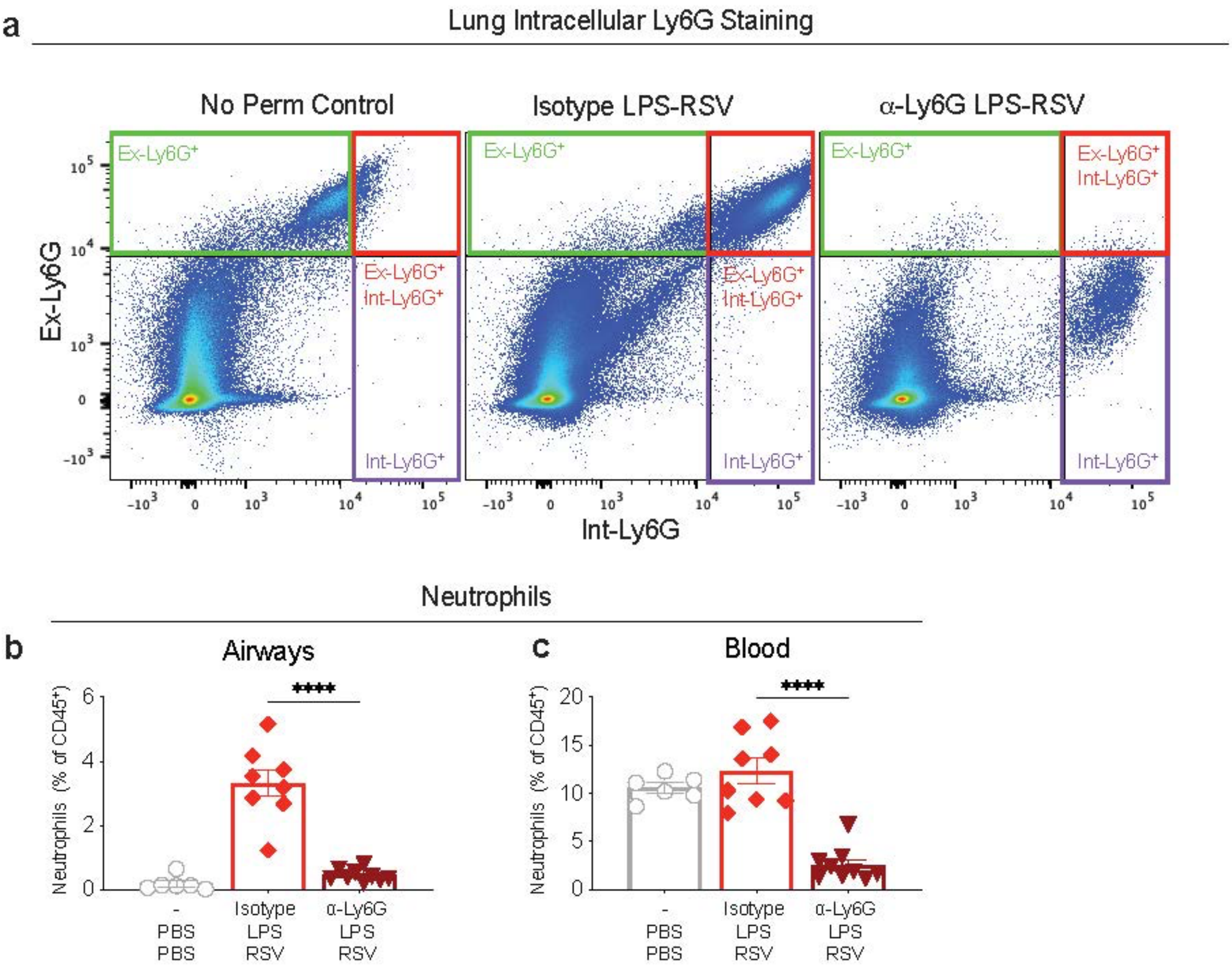

Supplementary Figure 6. Gating of neutrophils using intracellular Ly6G staining. a) Flow cytometry plots generated in FlowJo software showing expression of extracellular Ly6G (ex-Ly6G) staining versus intracellular Ly6G (int-Ly6G) on lung cells from isotype or α-Ly6G treated mice compared to non-permeabilised control lung cells from isotype mouse. Each plot represents one mouse/sample. Percentage of neutrophils (gated as intracellular Ly6G$^+$ cells) from total CD45$^+$ cells in the, **b)** airways and **c)** blood of α-Ly6G treated mice compared to isotype controls. Data are pooled from 2 experiments. PBS n=6; Isotype LPS-RSV n=8; α-Ly6G LPS-RSV n=9. For bar graphs, error bars represent SEM. A one-way ANOVA with multiple comparisons test was carried out to compare α-Ly6G LPS-RSV group with isotype LPS-RSV controls. Asterisks represent the p value compared to isotype controls; *≤0.05, **≤0.01, ***≤0.001, ****≤0.0001.

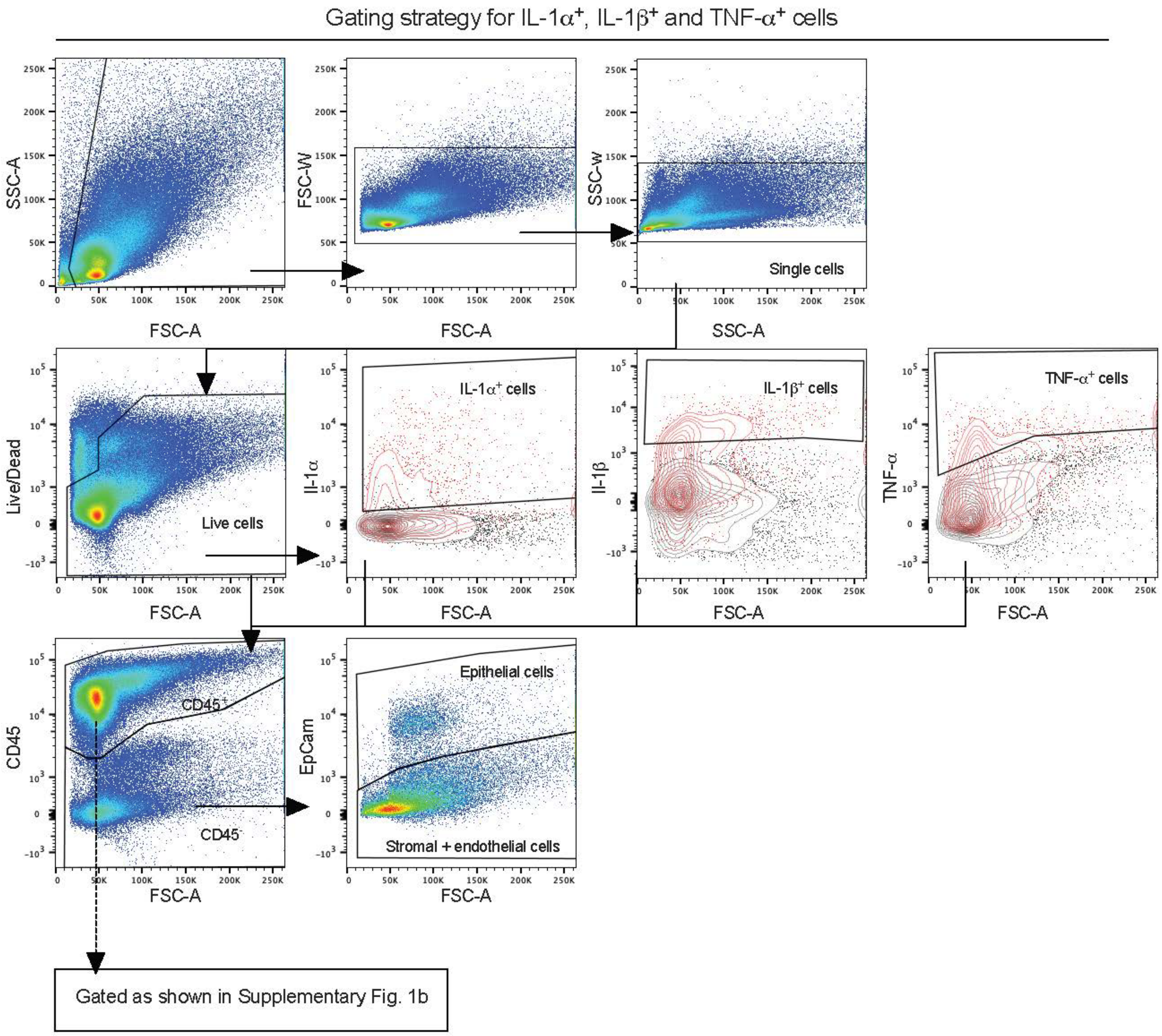

Supplementary Figure 7. Gating strategy for total IL-1α$^+$, IL-1β$^+$ and TNF-α$^+$ cells in the lung. Example flow cytometry plots showing lung cells from an LPS exposed mouse demonstrating the gating strategy for total IL-1α$^+$, IL-1β$^+$ and TNF-α$^+$ cells. Cells were gated as single, live cells and cytokine$^+$ populations were gated based on the respective FMO controls (shown on plots in black). From cytokine$^+$ populations, cells were separated into CD45$^+$ and CD45$^-$ cells. CD45$^-$ cells were further gated using Epcam to identify epithelial cells and the remaining cells in the stromal and endothelial compartment. CD45$^+$ cells were gated as shown in Supplementary Figure 1.

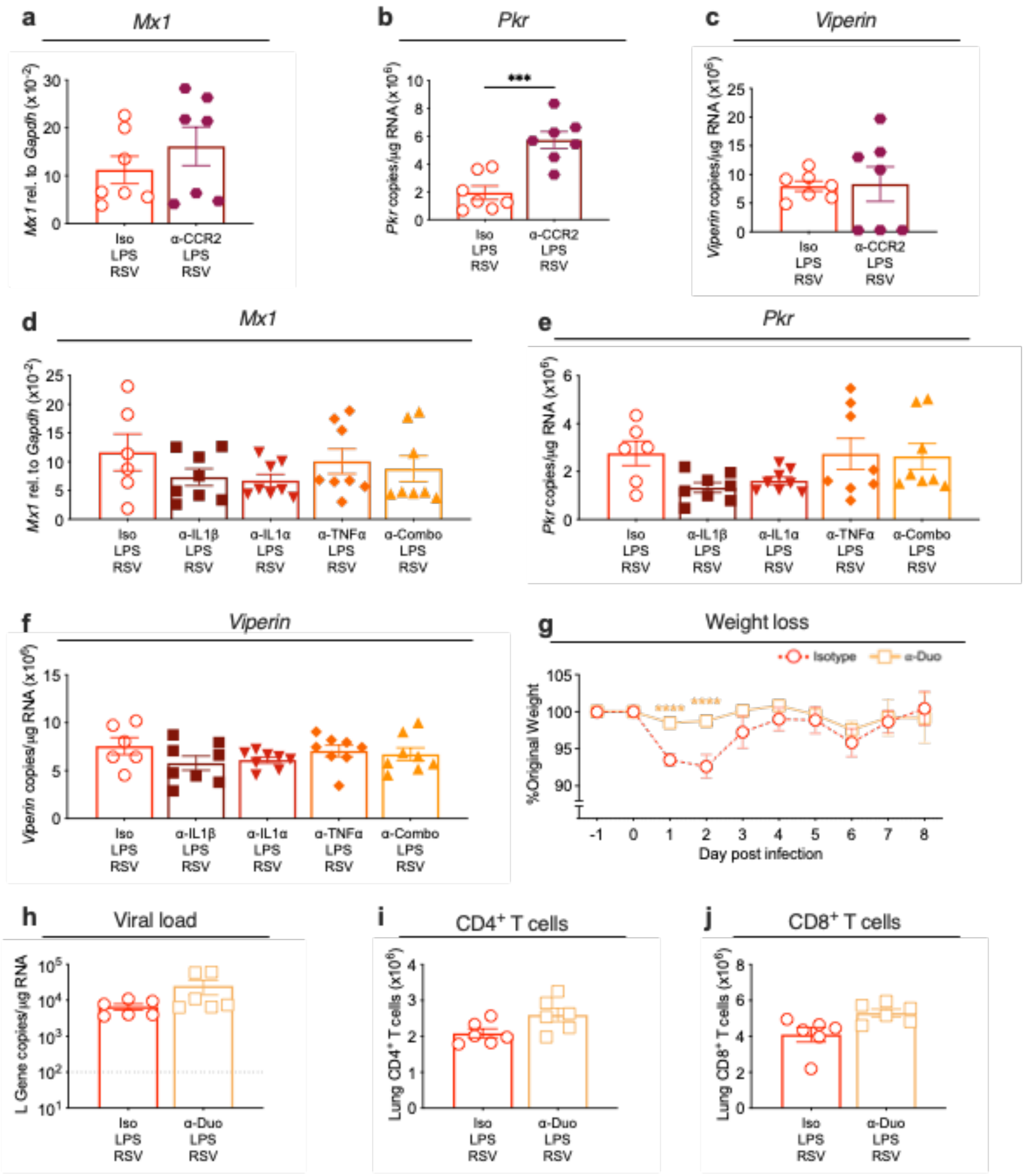

Supplementary Figure 8. IL-1α and TNF-α blockade had no effect on ISGs or the downstream T cell response and viral clearance. Gene expression of **a)** *Mx1,* **b)** *Pkr,* or **c)** *Viperin* on day 2 post infection in the lungs of mice treated with α-CCR2 antibodies. Gene expression of **d)** *Mx1,* **e)** *Pkr,* or **f)** *Viperin* on day 2 post infection in the lungs of mice treated with α-IL-1, α-IL-1β, α-TNF α or all three antibodies in combination. **g)** Daily weight loss up to day 8 post infection in LPS exposed, RSV infected mice treated with α-IL-1α and α-TNF-α in combination (α-Duo). **h)** Viral load in the

lungs of LPS exposed, RSV infected mice treated with α-Duo on day 8 post infection. Total numbers of **i)** $CD4^+$ T cells and **j)** $CD8^+$ T cells in the lungs of LPS exposed, RSV infected mice treated with α-Duo on day 8 post infection. For **a-c,** data are pooled from 2 experiments. α-CCR2 n=7; α-CCR2 isotype n=7. For **d-f**, data are pooled from 2 experiments. α-cytokine isotype LPS-RSV n=6; α-Combo isotype LPS-RSV n=4; α-IL-1α, α-IL-1β, α-TNF-α and α-Combo LPS-RSV n=8. For **g-j**, data are pooled from 2 experiments, n=6. Weight loss is plotted as the mean ± SEM. For bar graphs, error bars represent SEM. For weight loss, as data was paired, a two-way ANOVA with multiple comparisons test was carried out to compare depletion groups with isotype LPS-RSV controls. For flow cytometry and qPCR data, a one-way ANOVA with multiple comparisons test was carried out to compare depletion with isotype LPS-RSV controls. Asterisks represent the *p* value compared to isotype controls; *≤0.05, **≤0.01, ***≤0.001, ****≤0.0001.

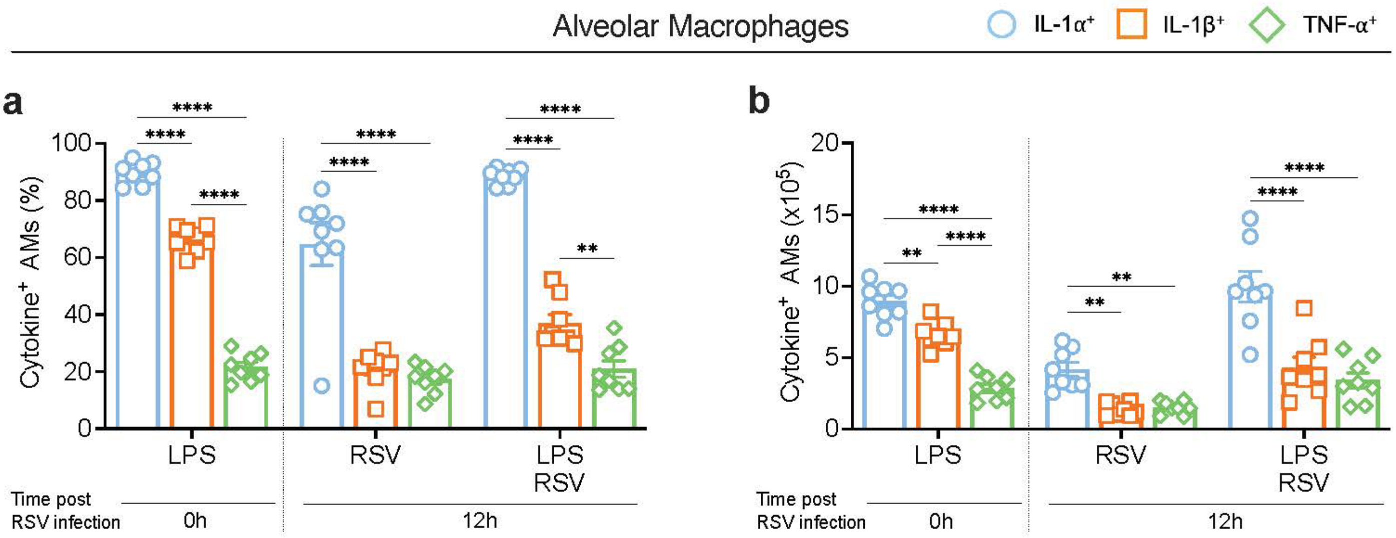

Supplementary Figure 9. IL-1α was the predominant cytokine produced by AMs. a) Percentage of total lung AMs that stained positive for IL-1α (blue), IL-1β (orange) and TNF-α (green), 12h after intranasal exposure to LPS, 12h after RSV infection or 12h after RSV infection in mice exposed to LPS 12h prior. **b)** Total number of IL-1α$^+$, IL-1β$^+$ and TNF-α$^+$ AMs in the lungs 12h after intranasal exposure to LPS, 12h after RSV infection or 12h after RSV infection in mice pre-exposed to LPS. Data are pooled from 2 experiments, n=8. Data are plotted as mean ± SEM. A two-way ANOVA was carried out to statistically compare each cytokine$^+$ population of AMs within each treatment group. Asterisks represent the *p* value compared to isotype controls; *≤0.05, **≤0.01, ***≤0.001, ****≤0.0001.
